# Safety and immunogenicity of a simian-adenovirus-vectored rabies vaccine: an open-label, non-randomised, dose-escalation, first-in-human, single-centre, phase 1 clinical trial

**DOI:** 10.1016/S2666-5247(22)00126-4

**Published:** 2022-07-27

**Authors:** Daniel Jenkin, Adam J Ritchie, Jeremy Aboagye, Sofiya Fedosyuk, Luke Thorley, Samuel Provstgaad-Morys, Helen Sanders, Duncan Bellamy, Rebecca Makinson, Zhi Quan Xiang, Emma Bolam, Richard Tarrant, Fernando Ramos Lopez, Abigail Platt, Ian Poulton, Catherine Green, Hildegund C J Ertl, Katie J Ewer, Alexander D Douglas

**Affiliations:** Jenner Institute University of Oxford, Oxford, UK; Centre for Clinical Vaccinology and Tropical Medicine, Churchill Hospital University of Oxford, Oxford, UK; Jenner Institute University of Oxford, Oxford, UK; Jenner Institute University of Oxford, Oxford, UK; Jenner Institute University of Oxford, Oxford, UK; Jenner Institute University of Oxford, Oxford, UK; Jenner Institute University of Oxford, Oxford, UK; Jenner Institute University of Oxford, Oxford, UK; Jenner Institute University of Oxford, Oxford, UK; Jenner Institute University of Oxford, Oxford, UK; Wistar Institute of Anatomy & Biology, Philadelphia, PA, USA; Clinical Biomanufacturing Facility University of Oxford, Oxford, UK; Clinical Biomanufacturing Facility University of Oxford, Oxford, UK; Centre for Clinical Vaccinology and Tropical Medicine, Churchill Hospital University of Oxford, Oxford, UK; Centre for Clinical Vaccinology and Tropical Medicine, Churchill Hospital University of Oxford, Oxford, UK; Centre for Clinical Vaccinology and Tropical Medicine, Churchill Hospital University of Oxford, Oxford, UK; Clinical Biomanufacturing Facility University of Oxford, Oxford, UK; Wistar Institute of Anatomy & Biology, Philadelphia, PA, USA; Jenner Institute University of Oxford, Oxford, UK; Jenner Institute University of Oxford, Oxford, UK

## Abstract

**Background:**

Rabies kills around 60 000 people each year. ChAdOx2 RabG, a simian adenovirus-vectored rabies vaccine candidate, might have potential to provide low-cost single-dose pre-exposure rabies prophylaxis. This first-in-human study aimed to evaluate its safety and immunogenicity in healthy adults.

**Methods:**

We did a single-centre phase 1 study of ChAdOx2 RabG, administered as a single intramuscular dose, with non-randomised open-label dose escalation at the Centre for Clinical Vaccinology and Tropical Medicine, Oxford, UK. Healthy adults were sequentially allocated to groups receiving low (5 × 10^9^ viral particles), middle (2·5 × 10^10^ viral particles), and high doses (5 x 10^10^ viral particles) of ChAdOx2 RabG and were followed up to day 56 after vaccination. The primary objective was to assess safety. The secondary objective was to assess immunogenicity with the internationally standardised rabies virus neutralising antibody assay. In an optional follow-up phase 1 year after enrolment, we measured antibody maintenance then administered a licensed rabies vaccine (to simulate post-exposure prophylaxis) and measured recall responses. The trial is registered with ClinicalTrials.gov, NCT04162600, and is now closed to new participants.

**Findings:**

Between Jan 2 and Oct 28, 2020, 12 adults received low (n=3), middle (n=3), and high doses (n=6) of ChAdOx2 RabG. Participants reported predominantly mild-to-moderate reactogenicity. There were no serious adverse events. Virus neutralising antibody concentrations exceeded the recognised correlate of protection (0·5 IU/mL) in three middle-dose recipients and six high-dose recipients within 56 days of vaccination (median 18·0 IU/mL). The median peak virus neutralising antibody concentrations within 56 days were 0·7 IU/mL (range 0·0–54·0 IU/mL) for the low-dose group, 18·0 IU/mL (0·7–18·0 IU/mL) for the middle-dose group, and 18·0 IU/mL (6·0–486·0 IU/mL) for the high-dose group. Nine participants returned for the additional follow-up after 1 year. Of these nine participants, virus neutralising antibody titres of more than 0·5 IU/mL were maintained in six of seven who had received middle-dose or high-dose ChAdOx2 RabG. Within 7 days of administration of the first dose of a licensed rabies vaccine, nine participants had virus neutralising antibody titres of more than 0·5 IU/mL.

**Interpretation:**

In this study, ChAdOx2 RabG showed an acceptable safety and tolerability profile and encouraging immunogenicity, supporting further clinical evaluation.

**Funding:**

UK Medical Research Council and Engineering and Physical Sciences Research Council.

## Introduction

Rabies virus causes a lethal encephalitis, which is estimated to be responsible for around 60 000 deaths per year, even though effective vaccines have been available for more than a century.^1^ This situation persists due to human and animal health system weaknesses, costs of licensed vaccines, and the requirement for multiple doses.

Dog vaccination is widely regarded as a highly cost-effective option for prevention of human rabies and is the backbone of the Global Alliance for Rabies Control’s Zero by 30 ambition to eliminate dog-transmitted human rabies by 2030.^2^ However, the programme faces substantial challenges in securing adequate resourcing and political commitment.

All rabies vaccines licensed for human use are composed of inactivated rabies virus. At least two doses are recommended in the context of pre-exposure prophylaxis (PrEP).^3^ Following a high-risk animal bite in an unvaccinated individual, receiving post-exposure prophylaxis (PEP) requires three clinic visits as an emergency. PEP vaccination should be initiated within 24 h of exposure but is often unavailable in local health facilities in rabies-endemic areas. The cost of vaccination and repeated travel are both factors hindering vaccine access. In addition to vaccination, rabies immune globulin is desirable for PEP after high-risk exposure, but is prohibitively expensive and rarely available in many rabies-endemic areas.^3,4^

In the event of a possible rabies virus exposure, benefits of having previously received PrEP include reduction in the number of PEP doses required and their urgency, avoidance of the need for rabies immune globulin, and the possibility of PrEP-mediated protection even in the absence of any PEP. However, PrEP is not a routine part of childhood vaccination schedules in most rabies-endemic countries.^5^ Under assumptions based on the cost of existing products and comparison, PrEP is only regarded as being a cost-effective option in exceptional circumstances, despite the fact that lifetime risk of death due to rabies exceeds one in 1000 across large areas of Africa and Asia.^1,4,6^ Improved access to PEP is typically regarded as a more cost-effective option than PrEP, but the delivery of urgent PEP in remote and unstable settings might prove programmatically challenging.^4,6^

Routine rabies PrEP with existing vaccines has been highly effective in reducing human rabies deaths in the Peruvian Amazon, where bats are the major vector, making control of the animal reservoir difficult or impossible.^6^ To increase the feasibility and cost-effectiveness of this strategy in other settings, we are developing ChAdOx2 RabG, a simian adenovirus vector encoding the rabies virus glycoprotein. We have previously reported that ChAdOx2 RabG had robust immunogenicity in mice.^7^ In a non-human primate study of a very similar product (AdC68rab.gp), administration of a single dose of 5 × 10^9^ viral particles (one tenth of a typical human dose of an adenovirus-vectored vaccine) resulted in 100% protection against a stringent rabies challenge 22 months after vaccination.^8^ The ChAdOx2 vector is based on a different adenovirus serotype from the ChAdOx1 nCoV-19 COVID-19 vaccine (Vaxzevria, Oxford/AstraZeneca) but can be produced with the same low-cost manufacturing process.^9,10^ Our main goal in developing this product is to enable low-cost single-visit PrEP to be included in routine vaccination schedules in rabies-endemic areas. We have completed a phase 1 clinical trial to investigate the safety and immunogenicity of ChAdOx2 RabG in healthy UK adults.

## Methods

### Study design

The RAB001 study is an open-label, non-randomised, dose escalation, first-in-human, phase 1 clinical trial done at a single centre (Centre for Clinical Vaccinology and Tropical Medicine, University of Oxford, Oxford, UK) and sponsored by the University of Oxford. The study was approved by the UK Medicines and Healthcare Products Regulatory Agency (CTA 21584/0417/001-0001), and the UK National Health Service (NHS) South Central—Oxford A Research Ethics Committee (19/SC/0408). ChAdOx2 RabG use was authorised by the Genetic Modification Safety Committee (GM462.19.122) at the Oxford University Hospitals NHS Foundation Trust. Design of ChAdOx2 RabG has previously been described.^7^ In brief, the vaccine uses the ChAdOx2 vector backbone (based on the AdC68 serotype),^11^ and encodes the full-length glycoprotein of Evelyn-Rokitnicki-Abelseth strain of the rabies virus. For this study, ChAdOx2 RabG was manufactured in compliance with Good Manufacturing Practice at the University of Oxford Clinical Biomanufacturing Facility, using our previously reported process.^9^ The trial has been conducted in accordance with the principles of the Declaration of Helsinki and Good Clinical Practice. An independent local safety monitor provided safety oversight of the trial, including safety reviews as described below. The study protocol is available in the appendix (pp 13–78).

### Participants

Participants with no previous history of rabies vaccination were recruited from the Thames Valley, UK, with the use of ethically approved online advertising materials. Individuals were required to complete an online questionnaire covering key exclusion criteria and were then invited for a screening visit if eligible. Following written informed consent, they were assessed for full eligibility at this visit, during which a medical history, physical examination, urinalysis, and clinical blood tests were done. Confirmation of rabies vaccine status was done by medical interview. Additionally, a summary of medical history was obtained from the general practitioner of each volunteer before vaccination.

### Procedures and outcomes

Each participant received a single dose of ChAdOx2 RabG, administered intramuscularly into the deltoid. The study proceeded through dose escalation, with each participant in group 1 (n=3) receiving 5 × 10^9^ virus particles (low dose), followed by group 2 (n=3) receiving 2·5 × 10^10^ viral particles (middle dose), and group 3 (n=6) receiving 5 × 1010 viral particles (high dose). Enrolment was staggered to allow for interim safety reviews to be done 48 h after the vaccination of the first volunteer as a sentinel in each group, and 7 days after vaccination of the third volunteer in each group (thus preceding each dose escalation).

The primary objective of the study was to assess safety. Following vaccination, participants attended a core series of follow-up visits at the following nominal timepoints: day 2, 7, 14, 28, and 56. Participants were questioned for the occurrence of severe adverse effects at all timepoints. Vital signs were also recorded at all study visits. Clinical laboratory blood tests, including full blood count, liver function, renal function and electrolytes, were done at baseline, day 2, day 7, and day 28. Additionally, participants were also required to complete an online daily symptom diary for 28 days following vaccination, including an initial 7-day solicited symptom collection period. The local and systemic solicited symptoms were defined in the trial protocol (appendix pp 13–78).

The secondary objective was to assess immunogenicity with the internationally standardised rabies virus neutralising antibody assay. Blood samples for immunology assays were taken on day 0 and at days 7, 14, 28, and 56. Live rabies virus neutralising antibodies were measured in assays at the Wistar Institute, Philadelphia, PA, USA, in accordance with Good Clinical Laboratory Practice. The rapid fluorescent focus inhibition test method was used, as previously described.^12^ The assay was done on mouse neuroblastoma cells and used the rabies virus reference strain, CVS-11 (American Type Culture Collection reference VR959), and the WHO 6th International Reference Standard^13^ to derive titres expressed in IU/mL. Methods for all additional immunogenicity assays are provided in the appendix (pp 11–12).

In an optional extended follow-up phase of the study, all participants were offered the opportunity to receive three doses of a licensed inactivated rabies vaccine (Rabipur; Valneva, Saint-Herblain, France) approximately 1 year after receiving ChAdOx2 RabG (day 365 ± 60 days, henceforth referred to as day 365). We used full doses of Rabipur administered intramuscularly in accordance with the Summary of Product Characteristics.^14^ The second and third doses of Rabipur were administered 7 and 21 days after the first. Additional blood samples for immunology assays were taken before the first dose of Rabipur and 7, 14, and 28 days after (study nominal days 365, 372, 379, and 386). Blood samples were always taken before vaccination during visits in which a vaccine was also administered.

Rabipur provided a simulation of receipt of PEP, as might be sought by a previous ChAdOx2 RabG recipient with an animal bite. Studies of novel regimens with existing licensed rabies vaccines have used similar simulated PEP designs. Similar to those studies, we considered virus neutralising antibody titres of 0·5 IU/mL or more 7 days after initiation of simulated PEP to be the key indicator of an adequate response (the anamnestic response to PEP would be especially important in any previously vaccinated individuals in whom virus neutralising antibody titres were less than 0·5 IU/mL at day 365).^15,16^ We considered the attainment of this threshold 7 days after a single dose to be a more stringent goal than attainment of a similar response with a WHO-recommended PEP regimen including a second dose at day 3. The regimen of single-site intramuscular administration on days 0, 7, and 21 was selected to maximise volunteer benefit from participation in this phase of the study, independent of the immunogenicity of ChAdOx2 RabG, as it is a UK-recommended PrEP regimen (unlike any WHO-recommended regimen for PEP in previously vaccinated individuals).^17^

Following the enrolment of the first nine participants in the study, recruitment was paused due to restrictions associated with the COVID-19 pandemic. These restrictions also resulted in some participants not being able to attend either their day 28 (one from group 3) or day 56 (three from group 2 and two from group 3) clinic visits. Core safety data, excluding blood testing, were collected remotely in these instances. Electronic data capture and clinical data management were carried out with OpenClinica (version 3.1). The study has been amended to include an extension phase in which additional participants have been recruited (not reported here) and remain under follow up. Data from this extended study will not be complete until 2023. Here, we report the data obtained from the original unextended design (appendix pp 14–78).

The trial is registered with ClinicalTrials.gov, NCT04162600, and is now closed to new participants.

### Role of the funding source

The authors designed, executed, analysed, and reported the study. The funders had no role in these activities other than review of the proposed study design during the funding application.

## Results

**Figure 1:**
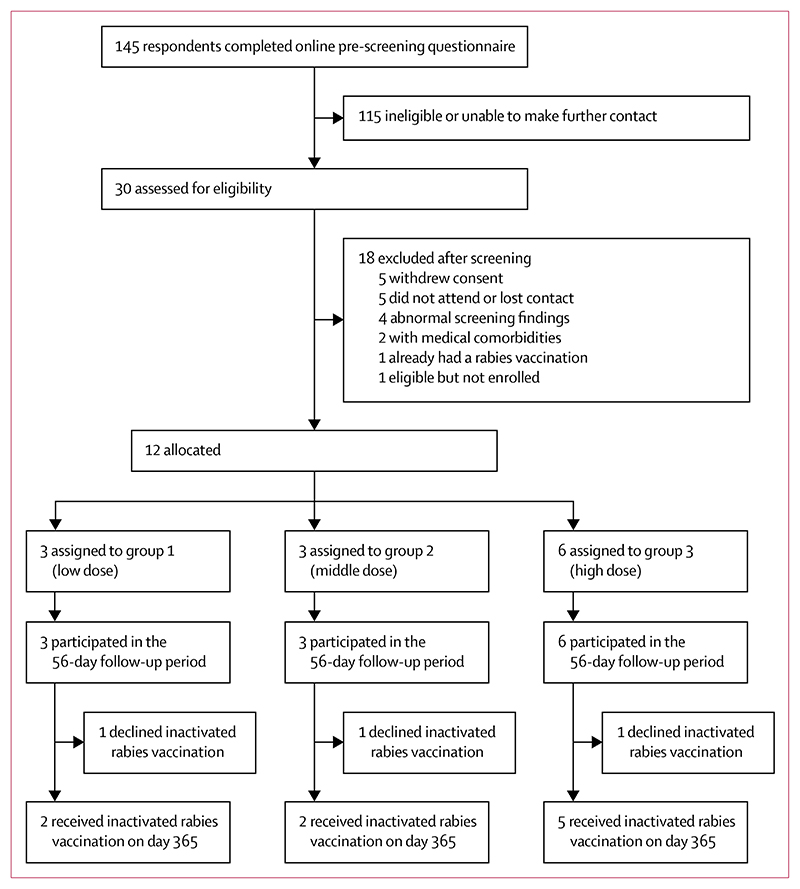
Trial profile

Participants were enrolled between Jan 2 and Oct 28, 2020. Following screening, participants underwent open-label non-randomised (sequential) allocation to groups 1–3 (figure 1). Baseline characteristics of the participants in each group are reported in the table. Overall, six men and six women were recruited, and the median age was 30 years (range 20–63). One clinic visit on day 28 and five clinic visits on day 56 were disrupted by local restrictions on clinical trial visits (on grounds of staff and volunteer safety and resource availability) due to the COVID-19 pandemic in March and April, 2020. In these cases, volunteers were contacted by phone to collect core safety data, but samples could not be collected for immunological assays or (in the case of the day 28 visit) clinical laboratory assays.

**Figure 2:**
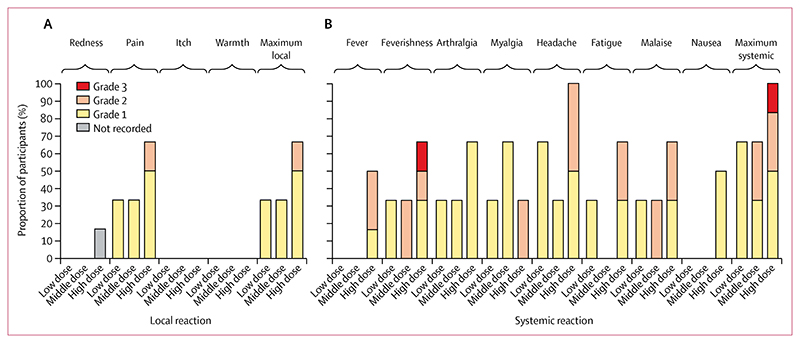
Solicited adverse events following vaccination with ChAdOx2 RabG For each of the individual-solicited local (A) and systemic (B) reactions, the maximum severity reported by each volunteer over the 7 days after vaccination is shown. In addition, to provide a global view of reactogenicity, the highest graded of all local and all systemic reactions is shown for each volunteer.

No serious adverse events or reactions occurred during the trial. Local reactogenicity was limited to predominantly mild (grade 1) injection site pain, primarily occurring within the high-dose group (figure 2A). No other local reactions were reported. As seen in previous trials and clinical use of other simian adenovirus vectored vaccines (including the one previous study^18^ of a ChAdOx2-vectored vaccine), mild to moderate systemic reactogenicity was common (figure 2B),^18,19^ with all participants in the middle-dose and high-dose groups reporting at least one systemic symptom. 50% of participants (including four of six in the high-dose group) reported use of antipyretic medication within 7 days of vaccine administration (appendix p 7). As with similar products, systemic reactogenicity was brief and self-limiting, occurring and resolving typically within 1–2 days after vaccination (appendix p 3). One volunteer in the high-dose group reported transient grade 3 feverishness (preventing daily activity) at day 1 after vaccination, which completely resolved by day 2. No other severe (grade 3 or more) adverse events or reactions were observed in the study.

Complete lists of recorded unsolicited adverse events and laboratory abnormalities are provided in the appendix (pp 7–8). Transient lymphopenia and neutropenia graded as mild or moderate were observed at day 2 for one (33%) of three participants in the low-dose group, zero (0%) of three in the middle-dose group, and three (50%) of six in the high-dose group. All laboratory adverse events resolved by day 7 without further investigation or intervention, and were judged not to be clinically significant.

Day 28 blood samples were collected for immunogenicity analysis from 11 of 12 volunteers, and day 56 samples for seven of 12 volunteers (including the single participant for whom no day 28 sample was collected). Nine of 12 volunteers returned for the optional additional follow up at days 365–386.

**Figure 3:**
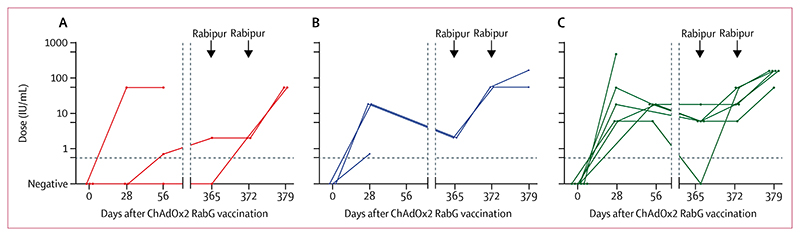
Rabies virus neutralising antibody responses Virus neutralising antibody responses at each measured timepoint are shown for group 1 (low dose, A), group 2 (middle dose, B) and group 3 (high dose, C). Arrowheads indicate administration of Rabipur (an inactivated rabies vaccine), with samples having been collected before Rabipur administration on applicable days. Each datapoint represents an individual volunteer, with lines connecting datapoints from an individual. Horizontal dashed line indicates 0·5 IU/mL (indicator of adequate vaccination). The same data are in the appendix (p 10).

**Figure 4:**
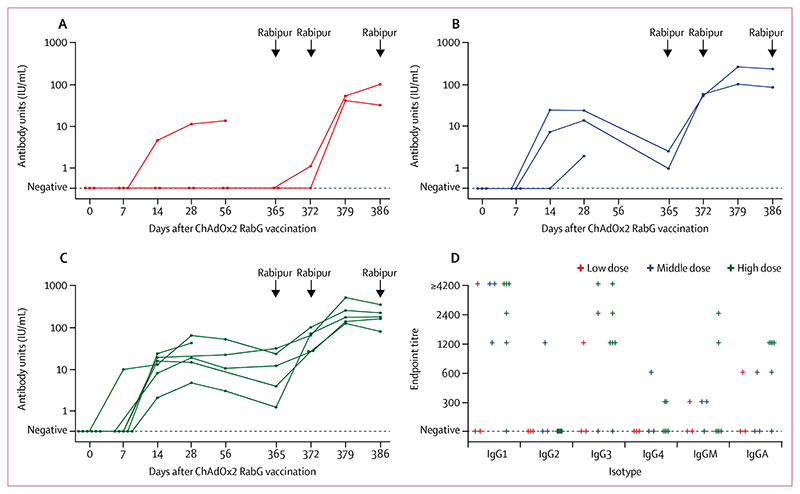
Rabies glycoprotein-binding antibody responses Total glycoprotein-binding IgG responses at each measured timepoint are shown for group 1 (low dose, A), group 2 (middle dose, B), and group 3 (high dose, C). Arrowheads indicate administration of Rabipur (an inactivated rabies vaccine), with samples having been collected before Rabipur administration on applicable days. Each datapoint represents an individual volunteer, with lines connecting datapoints from an individual. Endpoint titres of glycoprotein-binding immunoglobulin isotypes and subclasses at day 28 after administration of ChAdOx2 RabG are shown (D). Each datapoint represents an individual volunteer.

At enrolment, none of the volunteers had detectable rabies virus neutralising antibodies or rabies virus glycoprotein-binding antibodies, as assessed by ELISA (figure 3, figure 4). By day 56 after vaccination, 11 of 12 participants had attained virus neutralising antibody titres exceeding the value of 0·5 IU/mL, which signifies a satisfactory response to vaccination, with the exception being a single volunteer in the low-dose group (figure 3).^20,21^ The median peak virus neutralising antibody values from the timepoints available for analysis up to day 56 were 0·7 IU/mL (range 0·0–54·0 IU/mL) for the low-dose group, 18·0 IU/mL (0·7–18·0 IU/mL) for the middle-dose group, and 18·0 IU/mL (6·0–486·0 IU/mL) for the high-dose group. Seven of nine volunteers assessed at day 365 had virus neutralising antibody titres remaining at more than 0·5 IU/mL at this point (1 of 2 in the low-dose group, 2 of 2 in the middle-dose group, and 4 of 5 in the high-dose group): the two exceptions were the low-dose recipient who had not seroconverted after primary vaccination, and one high-dose recipient. The median virus neutralising antibody titre among the middle-dose and high-dose recipients on day 365 was 6·0 IU/mL (range 0·0–18·0).

All nine volunteers who received simulated PEP with licensed rabies vaccine (Rabipur) given intramuscularly on days 365 and 372 mounted prompt recall responses. All had virus neutralising antibody titres of more than 0·5 IU/mL by day 372 (ie, after a single simulated post-exposure dose). In some volunteers with virus neutralising antibody titres that had already exceeded 0·5 IU/mL by day 365, increase from the day 365 value was only apparent at day 379.

Total rabies glycoprotein-binding IgG kinetics, as measured by ELISA (figure 4), broadly mirrored virus neutralising antibody titres. Indeed, individual datapoints correlated closely with virus neutralising antibody results (Spearman’s *r*=0·89, 95% CI 0·81–0·94, across 45 samples for which both ELISA and virus neutralising antibody data were available; appendix p 4). Isotype and subclass ELISA showed that most volunteers had clear IgG1 and IgG3 responses, with weak IgG2 and IgG4 responses (figure 4D). This is consistent with the T-helper-1-skewed response induced by other adenovirus-vectored vaccines.^22^

At day 14 after primary vaccination, an antigen-specific interferon-gamma-producing T-cell response was detectable by ex vivo ELIspot in peptide-stimulated peripheral blood mononuclear cells (appendix p 5). There was a trend towards a dose–response relationship, with progressively stronger responses across group 1 (median 90 spot-forming cells per million peripheral blood mononuclear cells, range 54–384), group 2 (median 354, range 232–768), and group 3 (median 761, range 66–1509). Responses waned over the year after ChAdOx2 RabG vaccination but were then boosted by Rabipur administration. To attempt to dissect CD4+ and CD8+ T-cell responses, we used flow cytometry with T-cell intracellular cytokine staining but, in contrast to the ELIspot that used fresh peripheral blood mononuclear cells, we used frozen cells for intracellular cytokine staining and few responses were detectable (appendix p 6).

## Discussion

The findings from this first-in-human study show that the candidate rabies vaccine ChAdOx2 RabG has an encouraging immunogenicity profile at middle and higher dose levels, and a reactogenicity profile suitable for further evaluation in larger clinical trials.

No serious adverse reactions occurred. There was a single report of transient grade 3 feverishness; all other adverse events were mild or moderate (grade 1–2) in severity. There was nonetheless appreciable reactogenicity, tending to increase with increasing dose. Reactogenicity was comparable to that observed in the only previous phase 1 study of another ChAdOx2 vectored vaccine, and in larger numbers of participants receiving ChAdOx1 nCoV-19 at the 5 × 10^10^ viral particle dose in the phase 1 and 2 trial of that product.^18,19^ Prophylactic paracetamol was found to reduce reactogenicity without affecting immunogenicity during evaluation of ChAdOx1 nCoV-19.^19^ No recommendation was made for or against prophylactic paracetamol in the present study. 50% of participants made use of antipyretics in response to symptoms following vaccination.

Reactogenicity of the 5 x 1010 viral particles dose of ChAdOx1 nCoV-19 has subsequently proven to be acceptable across over 1 billion recipients. We anticipate further use of ChAdOx2 RabG at the maximum tolerable dose in the target populations (adults and children in rabies-endemic countries, particularly in Africa and Asia). Given the small numbers in the current trial and the variation between populations and contexts in reactogenicity of other adenovirus-vectored vaccines,^19,23^ additional data from studies in the target populations will be required to guide the choice of dose (and any recommendation for use of prophylactic paracetamol) for use in those settings.

Vaccine-induced thrombosis with thrombocytopenia has occurred as a very rare but serious adverse reaction to adenovirus-vectored COVID-19 vaccines.^24^ In the absence of complete understanding of the mechanism of vaccine-induced thrombosis with thrombocytopenia, it is unclear whether this risk is likely to apply to another serotype of adenovirus, delivering a non-coronavirus antigen to a predominantly Asian, African, and Latin American target population.^25^ There are large areas in which effective pre-exposure rabies prophylaxis might have a number-needed-to-treat to prevent a rabies death of well under 10 000.^1^ This is considerably lower than even the highest estimates of incidence of vaccine-induced thrombosis with thrombocytopenia. Thus, in our view, vaccine-induced thrombosis with thrombocytopenia does not preclude the possibility of a strongly positive risk-to-benefit balance of ChAdOx2 RabG vaccination in populations at high risk of rabies.

The existence of a robust mechanistic immunological correlate of protection against rabies, based on the internationally standardised virus neutralising antibody assay, is of great value to vaccine developers. This marker of protection allows substantial encouragement to be drawn from the immunogenicity results reported here (despite the small numbers of participants) and allows cautious comparison to results obtained with other vaccines (despite the absence of a comparator group within the trial).

In the past 10 years, there has been substantial interest in the potential abbreviation of licensed rabies vaccine administration regimens. In 2018, WHO recommendations state that single-visit vaccination with current licensed vaccines will probably provide partial protection but should not be considered a complete course.^2^ This recommendation is based on studies that have mostly evaluated single-visit multisite intradermal vaccination.^16,26,27^ These studies have tended to show that most, but not all, participants attain virus neutralising antibody titres of more than 0·5 IU/mL after vaccination (with median virus neutralising antibody titres <10 IU/mL at day 14–35), followed by waning of virus neutralising antibody titres to a median of less than 0·5 IU/mL 1–2 years later. These previous data suggest clear room for improvement in the immunogenicity of single-visit vaccination, and results in the current study appear favourably comparable.

In a 2021 study in healthy adults of a lipid-nanoparticle-formulated non-nucleoside-modified mRNA rabies vaccine developed by CureVac (Tübingen, Germany), only a minority of participants attained virus neutralising antibody titres of more than 0·5 IU/mL at the highest tolerated dose,^28^ as compared with median day 28 virus neutralising antibody titres of 18·0 IU/mL in the current study’s middle-dose and high-dose groups. This mirrors observations with mRNA SARS-CoV-2 vaccines: although no head-to-head comparison with licensed adenovirus-vectored vaccines has been done, some consider the immunogenicity and efficacy of non-nucleoside-modified mRNA SARS-CoV-2 vaccines to have been disappointing.^29^ In contrast, nucleoside-modified mRNA SARS-CoV-2 vaccines have higher tolerable doses and excellent immunogenicity.^30,31^ In our view, clinical evaluation of a nucleoside-modified mRNA rabies vaccine is worthwhile. However, there might remain doubts about the suitability of mRNA vaccine technology for single-dose PrEP in rabies-endemic countries on grounds of cost, temperature stability (and hence practicality of distribution), and durability of antibody responses.^32^

To our knowledge, there has been little characterisation of the cellular immune response to licensed rabies vaccines. Our data here show induction of T-cell responses similar to those seen with other adenovirus-vectored vaccines. The IgG1-skewed humoral response we observed here reflects the tendency of adenovirus vectors to induce a T-helper-1-skewed CD4+ T-cell response, and would be expected to mediate Fc-receptor-mediated and complement-mediated functionality. Although we did not directly measure memory B-cell responses, the anamnestic response seen here after simulated PEP suggests such responses are induced.

ChAdOx2 RabG thus induces multiple immune effectors that might contribute to the protection against viral infections by mechanisms additional to pre-formed virus neutralising antibodies. Although virus neutralising antibody titres of more than 0·5 IU/mL are accepted as a correlate of the robust protection induced by rabies vaccines, evidence from animal studies shows substantial (although not 100%) efficacy that can persist despite the waning of virus neutralising antibody concentrations to less than 0·5 IU/mL several years after vaccination. In an analysis combining multiple studies, approximately 80% of 492 dogs with undetectable prechallenge virus neutralising antibody titres (<0·03 IU/mL) 1 year after vaccination were protected against a stringent challenge to which 100% of unvaccinated controls succumbed.^21^ Caution is clearly required in extrapolation to humans from such animal studies, and the 0·5 IU/mL threshold remains attainable and appropriate to provide 100% protection after a suspected rabies exposure. Nonetheless, the use of the stringent threshold of 0·5 IU/mL to infer levels of protection might substantially underestimate the public health benefit, which might be achieved by mass pre-exposure vaccination with ChAdOx2 RabG, or indeed other rabies vaccines.

Our study has several limitations, some of which are common in first-in-human vaccine trials. The number of participants was low, and the participants were not drawn from the target population. As our main interest is in the performance of the new candidate relative to current licensed rabies vaccines in African and Asian children and adults, and the main objective of the current study was to gather sufficient safety data to support a further phase 1 study in a rabies-endemic area, we elected not to include a comparator group in the current study. Although the additional data we gathered here at 1 year after vaccination are of value, study of the longer-term maintenance of the response induced by ChAdOx2 RabG will now be necessary. We have not yet directly investigated the question of whether previous receipt of an adenovirus-vectored COVID-19 vaccine might attenuate the immune response to ChAdOx2 RabG, although few young children in rabies-endemic areas have received COVID-19 vaccines, and the available data suggest small induction of cross-serotype-neutralising antibody after vaccination with species E simian adenoviruses.^18^

Nonetheless, we believe this might be the most positive clinical data to date for a novel single-dose human rabies vaccine. As well as adenovirus-vectored vaccines’ clinical track record of safety, immunogenicity, and efficacy, the platform offers low manufacturing costs and stability suitable for straightforward distribution in rabies-endemic countries.^10,33^ In addition to the liquid formulations used with current licensed rabies vaccines, which permit storage at 2–8°C, we showed stability of ChAdOx2 RabG for 1 year at 20°C in a first-generation lyophilised formulation.^34^ The safety and immunogenicity of ChAdOx2 RabG are now being evaluated in a phase 1b–2 study in Tanzania.

## Supplementary Material

Supplementary materials

## Figures and Tables

**Table:** Baseline characteristics

	Group 1, 5 × 10^9^ viral particles (n=3)	Group 2, 2·5 × 10^10^ viral particles (n=3)	Group 3, 5 × 10^10^ viral particles (n=6)	All groups (n=12)
Sex				
Female	1 (33%)	2 (67%)	3 (50%)	6 (50%)
Male	2 (67%)	1 (33%)	3 (50%)	6 (50%)
Age, years	34 (23-53)	24 (20-47)	35 (21-63)	30 (20-63)
Ethnicity				
Asian or Asian British (Indian)	1 (33%)	¨	¨	1 (8%)
Asian or Asian British (other)	¨	1 (33%)	1 (17%)	2 (17%)
White (British)	2 (67%)	1 (33%)	5 (83%)	8 (67%)
White (other)	¨	1 (33%)	¨	1 (8%)

Data are n (%) or median (range).

## Data Availability

Deidentified participant data will be made available upon requests directed to the chief investigator. Proposals will be reviewed and approved by the sponsor, chief investigator, and collaborators on the basis of scientific merit. After approval of a proposal, data can be shared through a secure online platform after signing a data access agreement.

## Research in context

### Evidence before this study

To identify other novel rabies vaccine candidates, we searched PubMed with the following search terms: ((viral vector) OR (phase 1 OR phase i)) AND (rabies vaccine) AND (clinical trial). No time or language restrictions were used. Five studies were identified covering three vaccine candidates: phase 1 clinical trials of two rabies glycoprotein (RabG)-encoding mRNA vaccine candidates using a protamine-based formulation (CV7201) and a lipid nanoparticle formulation (CV7202), respectively. Additionally, three trials of a canarypox-vectored vaccine (ALVAC-RG) were identified. Depending on the dose and route of administration, CV7201-induced virus neutralising antibodies above the 0·5 lU/mL threshold (which signifies adequate vaccination) in up to 83% of volunteers, but required multiple administrations using specialised delivery devices to meet this threshold. CV7202 was modestly immunogenic as a two-dose regimen but a single dose (at any dose level) did not induce rabies virus neutralising antibody titres above the adequate response threshold. The high dose was not well tolerated and required a temporary halt of the study. ALVAC-RG was shown to be well tolerated and immunogenic when administered as two doses with a 4-week interval but did not induce robust responses after the first dose and appeared less immunogenic than a licensed rabies vaccine.

### Added value of this study

This study is the first clinical trial of ChAdOx2 RabG, a novel simian adenovirus-vectored vaccine encoding rabies glycoprotein, administered as a single intramuscular dose. The vaccine was safe and well tolerated in this study. Using the internationally standardised rabies virus neutralising antibody assay, all participants receiving either the medium or higher dose of ChAdOx2 RabG exceeded virus neutralising antibody titres associated with adequate vaccination within 56 days. Rabies glycoprotein-specific cellular immune responses were detected following vaccination in all volunteers.

### Implications of all the available evidence

Although highly efficacious, inactivated rabies vaccines have limitations including need for dosing at two or more visits and high cost. Partly for these reasons, use of human pre-exposure prophylaxis vaccination is low in countries with high burdens of rabies disease. Rabies glycoprotein-based vaccines that use modern vaccine platform technologies might be able to overcome these limitations, enabling low-cost single-visit pre-exposure prophylaxis. This phase 1 study provides initial supportive evidence for this approach and for continuing further clinical studies of the ChAdOx2 RabG candidate vaccine.
