# Supplementary material for: Safety and immunogenicity of a simian-adenovirus-vectored rabies vaccine: an open-label, non-randomised, dose-escalation, first-in-human, single-centre, phase 1 clinical trial
Source: Lancet Microbe. Author manuscript; Available in PMC 2023 Jul 28. (PMC7614839; doi:10.1016/S2666-5247(22)00126-4)
Supplement: Supplementary materials [file EMS181595-supplement-Supplementary_materials.pdf]

Contents

Appendix 1: Supplementary Material 2

Supplementary Figures 3

Supplementary Figure 1: Time course of solicited adverse events 3

Supplementary Figure 2: Relationship of VNA and glycoprotein binding total IgG ELISA measurements . 4

Supplementary Figure 3: Rabies glycoprotein-specific T cell responses 5

Supplementary Figure 4: T-cell flow cytometry with intracellular cytokine staining 6

Supplementary Tables 7

Supplementary Table 1: Antipyretic use 7

Supplementary Table 2: Unsolicited adverse events 8

Supplementary Table 3: Laboratory abnormalities 9

Supplementary Table 4: Rabies virus neutralizing antibody titres 10

Supplementary Methods 11

Humoral immunogenicity assays 11

T cell immunogenicity assays 11

Supplementary References 12

Appendix 2: Clinical Trial Protocol 13

Appendix 3: Laboratory Adverse Event Severity Grading 79

Appendix 1: Supplementary Material

Supplementary Figures

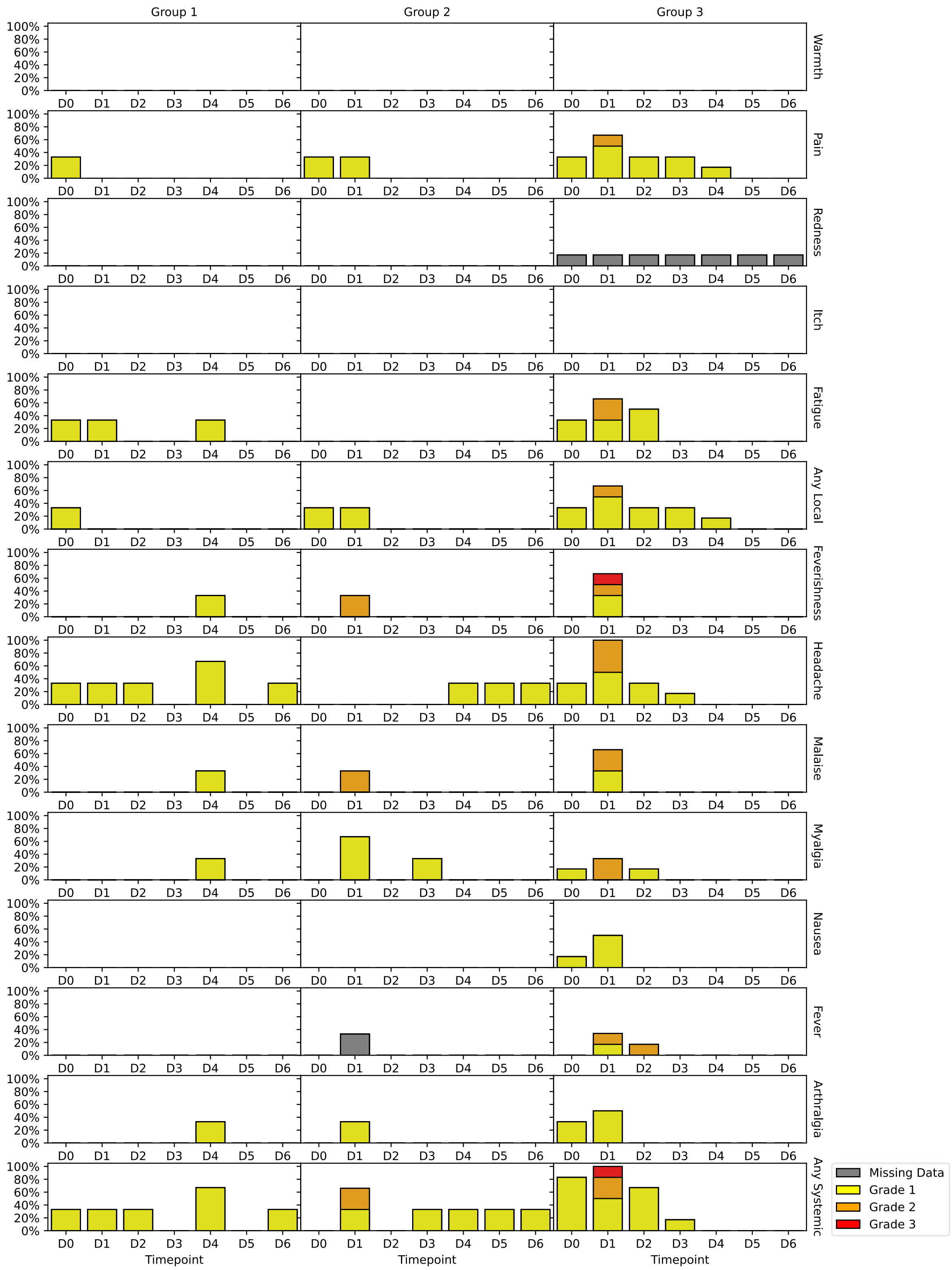

Supplementary Figure 1: Time course of solicited adverse events

Daily occurrence of solicited adverse events reported by participants within 7 days of vaccination for each group.

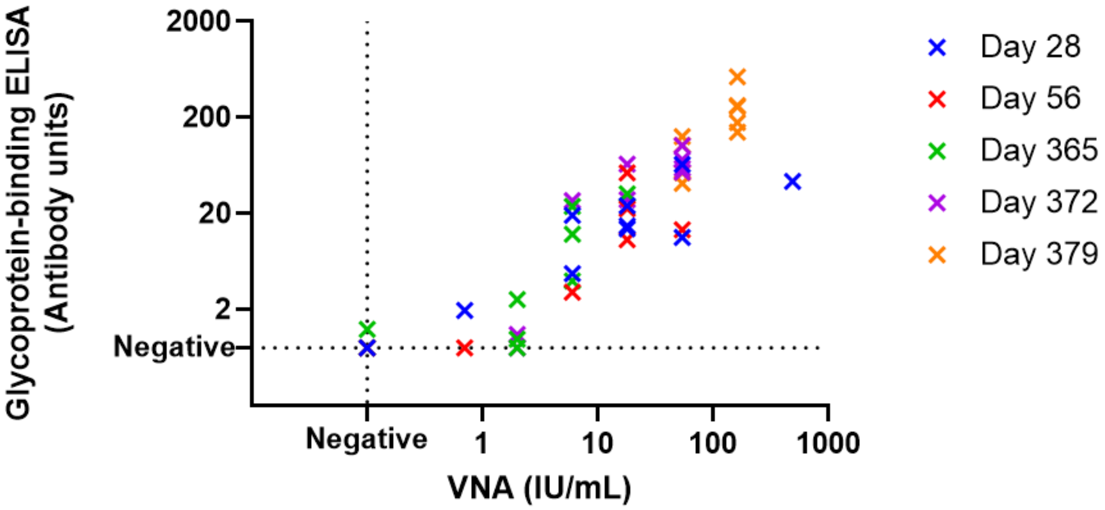

Supplementary Figure 2: Relationship of VNA and glycoprotein binding total IgG ELISA measurements
Data are shown for all 45 post-vaccination samples for which both VNA and ELISA data was obtained. Individual points are colour-coded by timepoint of collection, as indicated on the figure.

As stated in the text, across all samples for which both ELISA and VNA data available, ELISA results correlated closely with VNA results (Spearman's r=0.89, p<0.0001 for zero correlation, 95% confidence interval 0.81 – 0.94). Correlations were also statistically significant within individual timepoints (at day 28, r=0.81, p=0.004; at day 56, r=0.80, p=0.04; at day 365, r=0.86, p=0.005; at day 372, r=0.84, p=0.009; at day 379, r=0.87, p=0.02).

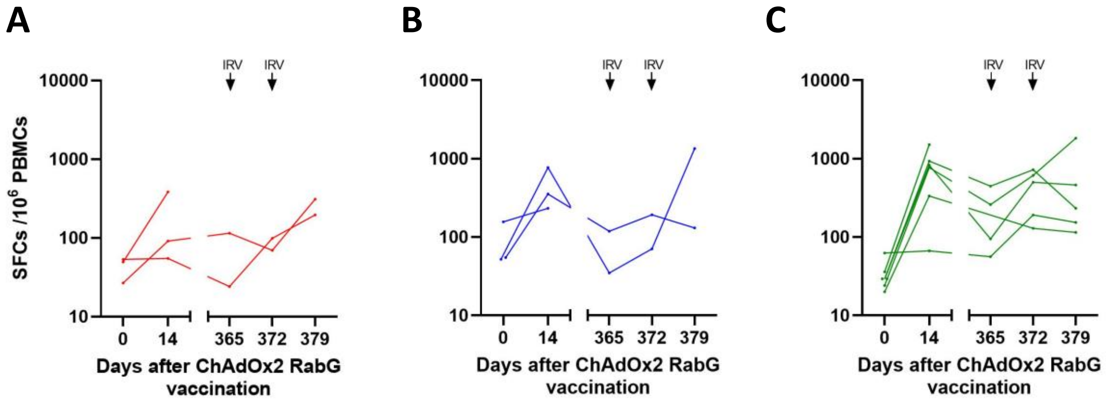

Supplementary Figure 3: Rabies glycoprotein-specific T cell responses
IFN-γ-producing T cell response measured by *ex vivo* ELISPOT in peptide-stimulated PBMCs are shown for group 1 (panel A), group 2 (panel B) and group 3 (panel C). Arrowheads indicate administration of Rabipur (IRV). Each data point represents an individual volunteer, with lines connecting data points from an individual. ELISpot responses shown are the sum of 5 peptide pools after subtraction of background (media only).

For all panels, group 1 (low dose) data are red, group 2 (mid dose) data are blue, and group 3 (high dose) data are green.

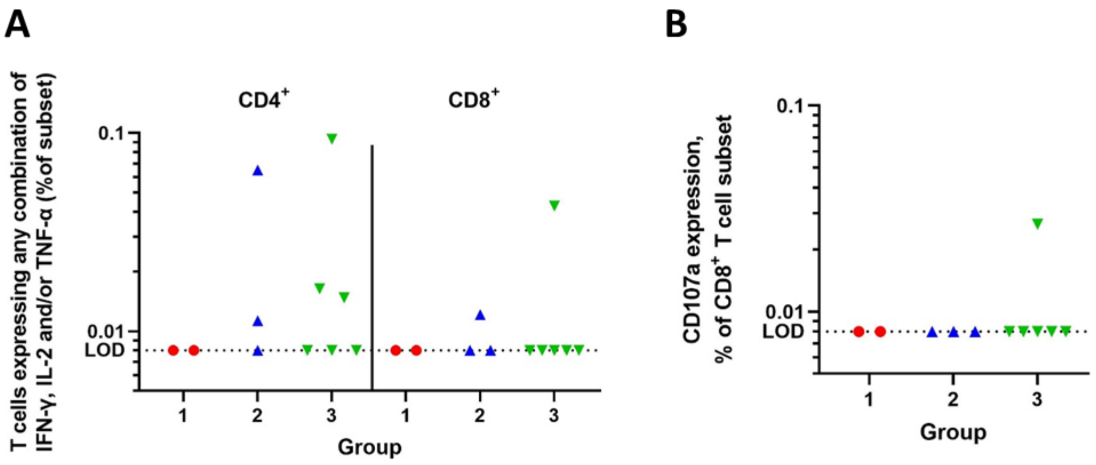

Supplementary Figure 4: T-cell flow cytometry with intracellular cytokine staining

Panel A: Scatterplot showing percentage of CD4+ and CD8+ cells expressing any combination of IFN-γ, IL-2 and/or TNF-α 14 days after ChAdOx2 RabG vaccination across the 3 groups.

Panel B: Scatterplot showing percentage of CD8+ cells expressing CD107a 14 days after ChAdOx2 RabG vaccination across the 3 groups (panel E).

In both panels, group 1 (low dose) data are red, group 2 (mid dose) data are blue, and group 3 (high dose) data are green. LOD; limit of detection.

Supplementary Tables

Supplementary Table 1: Antipyretic use

Self-reported antipyretic medication (paracetamol or NSAID use) due to vaccine related AEs within 7 days of ChAdOx2 RabG Vaccination.

	**Group 1 (n=3)**	**Group 2 (n=3)**	**Group 3 (n=6)**	**Total All groups (n=12)**
**Any antipyretic use n (%)**	1 (33%)	1 (33%)	4 (67%)	6 (50%)

Supplementary Table 2: Unsolicited adverse events

Related and unrelated unsolicited adverse events reported within 28 days of vaccination. Presented by MEDDRA System Organ Classes (SOC), MEDDRA Preferred Terms (PT) and severity grade.

	**Participants reporting AE, n (%)**			
	**Group 1 Low dose (n=3)**	**Group 2 Mid dose (n=3)**	**Group 3 High dose (n=6)**	**Total All groups (n=12)**
**AEs assessed as "possibly", "probably" or "definitely" related to vaccination**				
**General disorders and administration site conditions**	**–**	**1 (33%)**	**1 (17%)**	**2 (17%)**
Pyrexia				
Grade 1	–	–	1 (17%)	1 (8%)
Grade 2	–	1 (33%)	–	1 (8%)
Vaccination site pain				
Grade 1	–	–	1 (17%)	1 (8%)
**Nervous system disorders**	**–**	**–**	**2 (33%)**	**2 (17%)**
Dizziness				
Grade 1	–	–	1 (17%)	1 (8%)
Headache				
Grade 1	–	–	1 (17%)	1 (8%)
Typical aura without headache				
Grade 1	–	–	1 (17%)	1 (8%)
**Any unsolicited AE assessed as "Possibly", "Probably" or "Definitely" related**	**–**	**1 (33%)**	**2 (33%)**	**3 (25%)**
**AEs assessed as "No relationship" or "Unlikely related" to vaccination**				
**General disorders and administration site conditions**	**1 (33%)**	**1 (33%)**	**1 (17%)**	**3 (25%)**
Lethargy				
Grade 1	–	1 (33%)	–	1 (8%)
Grade 2	1 (33%)	–	–	1 (8%)
Procedural pain				
Grade 1	–	–	1 (17%)	1 (8%)
**Injury, poisoning and procedural complications**	**–**	**–**	**1 (17%)**	**1 (8%)**
Joint injury				
Grade 1	–	–	1 (17%)	1 (8%)
**Musculoskeletal and connective tissue disorders**	**–**	**1 (33%)**	**1 (17%)**	**2 (17%)**
Back pain				
Grade 1	–	–	1 (17%)	1 (8%)
Tendonitis				
Grade 2	–	1 (33%)	–	1 (8%)
**Nervous system disorders**	**2 (67%)**	**–**	**3 (50%)**	**5 (42%)**
Headache				
Grade 1	2 (67%)	–	3 (50%)	5 (42%)
Grade 2	1 (33%)	–	–	1 (8%)
**Respiratory, thoracic and mediastinal disorders**	**1 (33%)**	**1 (33%)**	**2 (33%)**	**4 (33%)**
Epistaxis				
Grade 1	–	–	1 (17%)	1 (8%)
Nasal congestion				
Grade 1	–	1 (33%)	–	1 (8%)
Oropharyngeal pain				
Grade 1	–	1 (33%)	1 (17%)	2 (17%)
Rhinorrhoea				
Grade 1	1 (33%)	1 (33%)	–	2 (17%)
Sneezing				
Grade 1	–	1 (33%)	–	1 (8%)
**Any unsolicited AE assessed as "No Relationship" or "Unlikely" related**	**3 (100%)**	**2 (67%)**	**5 (83%)**	**10 (83%)**

Supplementary Table 3: Laboratory abnormalities

Clinical laboratory results obtained measured at each evaluated timepoint in the trial for all groups. Abnormal results were graded according to a pre-specified laboratory adverse events severity grading scale (Appendix 3).

	Low dose			Mid dose			High dose		
	**D2**	**D7**	**D28**	**D2**	**D7**	**D28**	**D2**	**D7**	**D28**
**Albumin (n evaluated)**	**3**	**3**	**3**	**3**	**3**	**3**	**6**	**6**	**5**
Normal	2	3	3	3	3	3	6	6	5
Grade 1 Decreased Albumin	1	—	—	—	—	—	—	—	—
**Alkaline Phosphatase (n evaluated)**	**3**	**3**	**3**	**3**	**3**	**3**	**6**	**6**	**5**
Normal	3	3	3	3	3	3	6	6	5
**ALT (n evaluated)**	**2**	**3**	**3**	**3**	**3**	**3**	**6**	**6**	**5**
Normal	2	3	3	3	3	3	6	6	5
**Bilirubin (n evaluated)**	**3**	**3**	**3**	**3**	**3**	**3**	**6**	**6**	**5**
Normal	3	3	3	3	3	3	6	5	5
Grade 1 Increased Bilirubin	—	—	—	—	—	—	—	1	—
**Creatinine (n evaluated)**	**3**	**3**	**3**	**3**	**3**	**3**	**6**	**6**	**5**
Normal	3	3	3	3	3	3	6	6	5
**Potassium (n evaluated)**	**3**	**3**	**3**	**3**	**3**	**3**	**6**	**6**	**5**
Normal	3	3	3	3	3	3	6	6	5
**Sodium (n evaluated)**	**3**	**3**	**3**	**3**	**3**	**3**	**6**	**6**	**5**
Normal	3	3	3	3	3	3	6	6	5
**Urea (n evaluated)**	**2**	**3**	**3**	**3**	**3**	**3**	**6**	**6**	**5**
Normal	2	3	3	3	3	3	6	6	5
**Eosinophils (n evaluated)**	**3**	**3**	**3**	**3**	**3**	**3**	**6**	**6**	**5**
Normal	3	3	3	3	3	3	6	6	5
**Lymphocytes (n evaluated)**	**3**	**3**	**3**	**3**	**3**	**3**	**6**	**6**	**5**
Normal	2	3	3	3	3	3	4	6	5
Grade 1 Decreased Lymphocytes	1	—	—	—	—	—	1	—	—
Grade 2 Decreased Lymphocytes	—	—	—	—	—	—	1	—	—
**Neutrophils (n evaluated)**	**3**	**3**	**3**	**3**	**3**	**3**	**6**	**6**	**5**
Normal	3	3	3	3	3	3	3	6	5
Grade 1 Decreased Neutrophils	—	—	—	—	—	—	2	—	—
Grade 2 Decreased Neutrophils	—	—	—	—	—	—	1	—	—
**Platelets (n evaluated)**	**3**	**3**	**3**	**3**	**3**	**3**	**6**	**6**	**5**
Normal	3	3	3	3	3	3	6	6	5
**White Cell Count (n evaluated)**	**3**	**3**	**3**	**3**	**3**	**3**	**6**	**6**	**5**
Normal	3	3	2	3	3	3	3	6	5
Grade 1 Decreased WCC	—	—	1	—	—	—	2	—	—
Grade 2 Decreased WCC	—	—	—	—	—	—	1	—	—
**Haemoglobin (n evaluated)**	**3**	**3**	**3**	**3**	**3**	**3**	**6**	**6**	**5**
Normal	3	3	3	3	3	3	6	6	5

Supplementary Table 4: Rabies virus neutralizing antibody titres

Virus neutralizing antibody data, as presented in Figure 3. 'Neg' denotes negative. ‘Na’ denotes timepoints were VNA titres were not assessed. Samples were not tested at lesser dilutions than that corresponding to 0.7 IU/mL, with the exception of the sample for volunteer 3-2 on day 365, which was negative when tested at a dilution corresponding to 0.2 IU/mL.

Group	Volunteer Code	VNA Titre (IU/mL)					
		**D0**	**D28**	**D56**	**D365**	**D372**	**D379**
ChAdOx2 RabG 5x10^9	1-1	Neg	Neg	Neg	Neg	2	54
	1-1	Neg	Neg	0.7	2	2	54
	1-3	Neg	54	54	Na	Na	Na
ChAdOx2 RabG 2.5x10^{10}	2-1	Neg	18	Na	2	54	54
	2-2	Neg	0.7	Na	Na	Na	Na
	2-3	Neg	18	Na	2	54	162
ChAdOx2 RabG 5x10^{10}	3-1	Neg	6	18	6	18	162
	3-2	Neg	6	6	Neg	54	162
	3-3	Neg	486	Na	Na	Na	Na
	3-4	Neg	18	Na	6	6	54
	3-5	Neg	54	18	6	54	162
	3-6	Neg	Na	18	18	18	162

Supplementary Methods

Humoral immunogenicity assays

Rabies virus glycoprotein was expressed recombinantly by transient transfection of mammalian cells, using the Expi293 system (Thermo Scientific) in accordance with the manufacturers' instructions. The antigen is challenging to produce, and to achieve sufficient productivity we used a previously reported, well-expressed chimera of codon-optimised Pasteur virus strain glycoprotein ectodomain coding sequence with the SAD-B19 strain intravirion domain.[1] The construct also included a C-terminal C-tag.[2] Cells were harvested three days after transfection, pelleted by centrifugation, and subjected to detergent extraction using 1% β-octylglucoside (Generon Ltd). Rabies glycoprotein was then purified from the extract by affinity chromatography using C-tag resin (Thermo Scientific) followed by size-exclusion chromatography using Superose 6 10/300 Increase column (Cytiva) in buffer 50 mM HEPES pH 7.4, 150 mM NaCl, 1% β-octylglucoside. This resulted in an antigen with >90% purity (by Coomassie-stained SDS-PAGE gel) and a single peak on the size exclusion elution profile consistent with the expected size of trimeric glycoprotein. This antigen was used to coat ELISA plates at a concentration of 2 µg/mL.

Quantification of total rabies glycoprotein-binding IgG was performed as described previously, with the exception of change in the coating antigen.[3] As a positive reference sample, we used plasma for the day 28 visit of one participant in the high dose group. Results were expressed in arbitrary antibody units (AU), defined using this reference sample as previously described.[3]

Quantification of immunoglobulin isotypes and subclasses also used a previously described method,[4] with the exception of the change in the coating antigen and the use of a two-fold dilution-series / endpoint titer quantification approach. Reported titers are the greatest dilution at which the diluted sample resulted in optical density exceeding that of the mean plus three standard deviations of optical density among six pre-immune samples at dilutions of 1:600 (for IgG1, IgG2, or IgM), or 1:300 (for IgG3, IgG4 and IgA), and also exceeding the optical density of the individual participant's pre-immune sample at that dilution by >0.1 absorbance units.

T cell immunogenicity assays

Ex vivo interferon-γ ELISpot and intracellular cytokine staining (ICS) flow cytometry assays were performed as previously described,[5] with the exception that stimulation used 20-mer peptides with 10-mer overlaps, spanning the complete ERA strain rabies virus glycoprotein (Mimotopes).

For ELISpot, freshly isolated peripheral blood mononuclear cells (PBMCs) from days 0, 14, 365, 372 and 379 were stimulated, in triplicate, with 5 pools of 10 overlapping peptides each. The lower limit of detection for the ELISPOT assay was 20 spot-forming cells (SFC) per million PBMC.

ICS was performed from frozen aliquots of PBMCs from day 14, stimulated with a single pool containing all peptides spanning the antigen, with data from 1 participant in group 1 excluded due to lack of sufficient available cells. Data were analysed in FlowJo v10.7 (BD Life Sciences). The lower limit of detection for cytokines and CD107a in the ICS assay was 0.008% of the parent gate ($CD4^+$ or $CD8^+$).

Supplementary References

1. Kim EJ, Jacobs MW, Ito-Cole T, Callaway EM. Improved Monosynaptic Neural Circuit Tracing Using Engineered Rabies Virus Glycoproteins. *Cell Rep* 2016; **15**(4): 692-9.

2. De Genst EJ, Guilliams T, Wellens J, et al. Structure and properties of a complex of alpha-synuclein and a single-domain camelid antibody. *J Mol Biol* 2010; **402**(2): 326-43.

3. Miura K, Orcutt AC, Muratova OV, Miller LH, Saul A, Long CA. Development and characterization of a standardized ELISA including a reference serum on each plate to detect antibodies induced by experimental malaria vaccines. *Vaccine* 2008; **26**(2): 193-200.

4. Biswas S, Choudhary P, Elias SC, et al. Assessment of humoral immune responses to blood-stage malaria antigens following ChAd63-MVA immunization, controlled human malaria infection and natural exposure. *PLoS One* 2014; **9**(9): e107903.

5. Ewer K, Rampling T, Venkatraman N, et al. A Monovalent Chimpanzee Adenovirus Ebola Vaccine Boosted with MVA. *N Engl J Med* 2016; **374**(17): 1635-46.

Appendix 2: Clinical Trial Protocol

UNIVERSITY OF OXFORD

A phase I clinical trial to determine the safety and immunogenicity of the candidate rabies vaccine ChAdOx2 RabG in UK healthy adult volunteers

Study Reference: RAB001

Protocol Number: v1.1

Date: 26th March 2020

Chief Investigator: Dr Alexander D. Douglas

Sponsor: University of Oxford

Funder: UK Medical Research Council

REC Number: XXX

EudraCT Number: 2019-002800-41

IRAS Reference: 269046

©Oxford University

PAGE LEFT INTENTIONALLY BLANK

©Oxford University

**Full Study Title**	A phase I study to determine the safety and immunogenicity of the candidate rabies vaccine ChAdOx2 RabG in UK healthy adult volunteers.<br>**Study Code**: RAB001<br>**EudraCT Number**: 2019-002800-41<br>**IRAS project ID**: 269046
**Chief Investigator**	**Dr Alexander D. Douglas**<br>Jenner Institute<br>Wellcome Centre for Human Genetics<br>University of Oxford,<br>Roosevelt Drive, Headington<br>Oxford, OX3 7BN<br>Email: sandy.douglas@ndm.ox.ac.uk
**Trial Site**	**Centre for Clinical Vaccinology and Tropical Medicine**<br>Churchill Hospital, Old Road, Headington<br>Oxford, OX3 7LE
**Sponsoring Institution**	**University of Oxford**<br>Clinical Trials and Research Governance<br>Boundary Brook House. Churchill Drive. Headington<br>Oxford, OX3 7GB<br>Tel: 01865 616480<br>Email: ctrg@admin.ox.ac.uk
**External Monitor**	**Clinical Trials and Research Governance**<br>University of Oxford<br>Boundary Brook House. Churchill Drive. Headington<br>Oxford, OX3 7GB
**Local Safety Monitor (Chair of Local Safety Committee)**	**Dr Brian Angus**<br>Centre for Clinical Vaccinology and Tropical Medicine<br>Churchill Hospital, Old Road, Headington<br>Oxford, OX3 7LE<br>Tel: 01865 220289<br>Email: brian.angus@ndm.ox.ac.uk

©Oxford University

Confidentiality Statement

This document contains confidential information that must not be disclosed to anyone other than the Sponsor, the Investigator Team, host organisation, members of the Research Ethics Committee and relevant regulatory bodies. This information cannot be used for any purpose other than the evaluation or conduct of the clinical investigation without the prior written consent of Dr Alexander D. Douglas.

Statement of Compliance

The trial will be conducted in compliance with the protocol, the principles of Good Clinical Practice Guideline, Medicines for Human Use (Clinical Trials) Regulations 2004 (as amended) and all other applicable regulatory requirements.

Chief Investigator Approval, Agreement and Conflict of Interest statement

I have read the trial protocol and agree to conduct the trial in compliance with the protocol, the principles of Good Clinical Practice and all applicable regulatory requirements.

Dr Alexander Douglas is a named inventor on a patent application relating to the ChAdOx2 adenovirus serotype and would share in any income resulting from the patent.

Alexander D. Douglas		26th March 2020
**Chief Investigator Name**	**Signature**	**Date**

©Oxford University

Modification History

Version	Date	Author(s)	Modifications
0.1	25 June 2019	Alexander D. Douglas, Daniel Jenkin, Adam J. Ritchie	First draft
0.2	8 July 2019	Alexander D. Douglas, Adam J. Ritchie	Response to CTRG comments and insertion of additional sampling.
0.3	10 July 2019	Adam J. Ritchie	Response to CTRG comments
1.0	16 July 2019	Adam J. Ritchie	Update to versions 1.0 after full CTRG review
1.1	26 March 2020	Daniel Jenkin	Non-substantial amendment in response to ongoing COVID-19 public health crisis:<br>1. Option to carry out study follow up visits as home visits<br>2. Temporary pause on recruitment

©Oxford University

Table of Contents

1 SYNOPSIS 10

2 ABBREVIATIONS 12

3 BACKGROUND & RATIONALE 14
3.1 Rabies 14
3.2 Impact of rabies 14
3.3 Current rabies control and vaccination 14
3.3.1 Pre-exposure prophylaxis (PrEP) 15
3.3.2 Post-exposure prophylaxis (PEP) 15
3.3.3 Animal vaccination 15
3.4 The need for a new vaccine 15
3.5 Rabies glycoprotein as a vaccine antigen 16
3.6 Adenovirus-vectored vaccines 16
3.7 ChAdOx2 17
3.8 Development of ChAdOx2 vaccine vector 18
3.9 Development of ChAdOx2 RabG 19
3.10 Preclinical studies 19
3.11 Previous clinical experience 19
3.12 Rationale 22
3.13 Vaccine development strategy 22
3.14 Rationale for optional extended follow-up including immunisation with existing rabies vaccine 22
3.15 Rationale for optional saliva sample collection and analysis 23

4 OBJECTIVES AND ENDPOINTS 25
4.1 Primary Objective 25
4.1.1 Primary Outcome Measures 25
4.2 Secondary Objective 25
4.2.1 Secondary Outcome Measures 25
4.3 Additional (exploratory) Objectives 25

5 STUDY OVERVIEW 27
5.1 Rationale for Selected Doses 27
5.2 Study Groups 28
5.3 Vaccination and safety reviews 28
5.4 Duration of study 28
5.5 Definition of Start and End of Trial 28
5.6 Potential Risks for volunteers 29
5.7 Known Potential Benefits 29

6 RECRUITMENT AND WITHDRAWAL OF TRIAL VOLUNTEERS 30
6.1 Volunteers 30

©Oxford University

6.2 Informed consent ... 30
6.3 Inclusion and exclusion criteria ... 31
6.3.1 Inclusion criteria ... 31
6.3.2 Exclusion criteria ... 31
6.3.3 Exclusion criteria for optional follow-up ... 32
6.3.4 Effective contraception for female volunteers ... 33
6.3.5 Prevention of 'over volunteering' ... 33
6.3.6 Criteria for postponement of vaccination (Individual holding rules) ... 33
6.3.7 Withdrawal of volunteers ... 34
6.4 Compliance with dosing regime ... 34
6.5 Pregnancy ... 35

7 CLINICAL PROCEDURES ... 36
7.1 Study procedures ... 36
7.2 Observations ... 36
7.3 Blood, urine and saliva sampling and analysis ... 36
7.4 Study visits ... 38
7.4.1 Screening visit ... 38
7.4.2 Day 0: Enrolment and ChAdOx2 RabG vaccination visit ... 39
7.4.3 ChAdOx2 RabG vaccinations ... 39
7.4.4 Sequence of enrolment and ChAdOx2 RabG vaccination of volunteers ... 40
7.4.5 Subsequent visits during core study period: days 2, 7, 14, 28, 56 ... 41
7.4.6 Optional IRV vaccination and follow-up ... 41
7.4.7 Schedule of attendances ... 42

8 INVESTIGATIONAL PRODUCTS ... 43
8.1 Manufacturing and Presentation ... 43
8.1.1 ChAdOx2 RabG ... 43
8.1.2 IRVs ... 43
8.2 Supply ... 43
8.2.1 ChAdOx2 RabG ... 43
8.2.2 IRVs ... 44
8.3 Storage ... 44
8.3.1 ChAdOx2 RabG ... 44
8.3.2 IRVs ... 44
8.4 Administration of Investigational Medicinal Products ... 44
8.5 Minimising environmental contamination with genetically modified organisms (GMO) ... 44

9 ASSESSMENT OF SAFETY ... 46
9.1 Definitions ... 46
9.1.1 Adverse Event (AE) ... 46
9.1.2 Adverse Reaction (AR) ... 46

©Oxford University

9.1.3 Serious Adverse Event (SAE) 46
9.1.4 Serious Adverse Reaction (SAR) 47
9.1.5 Suspected Unexpected Serious Adverse Reaction (SUSAR) 47
9.2 Causality assessment 47
9.3 Expectedness assessment 48
9.4 Reporting procedures for all Adverse Events (see SOP VC027) 48
9.4.1 Reporting procedures for SAEs (see SOP OVC005 Safety Reporting)....................48
9.4.2 Reporting Procedures for SUSARS 49
9.4.3 Development Safety Update Report....................49
9.5 Assessment of severity 49
9.6 Procedures to be followed in the event of abnormal findings 50
9.7 Local Safety Monitor....................51
9.8 Interim safety reviews 51
9.9 Safety Holding Rules 52
9.9.1 Group holding rules 52
10 DATA MANAGEMENT....................54
10.1 Data Handling....................54
10.2 Record Keeping....................54
10.3 Source Data and Case Report Forms (CRFs) 54
10.4 Data Protection 54
10.5 Data Quality 55
11 STATISTICS....................56
11.1 Sample Size Selection....................56
12 ETHICS AND REGULATORY CONSIDERATIONS 57
12.1 Declaration of Helsinki....................57
12.2 Guidelines for Good Clinical Practice 57
12.3 Approvals 57
12.4 Reporting....................57
12.5 Volunteer Confidentiality....................58
13 QUALITY CONTROL AND QUALITY ASSURANCE PROCEDURES....................59
13.1 Investigator procedures 59
13.2 Monitoring 59
13.3 Protocol deviation 59
13.4 Audit & inspection 59
14 FINANCING AND INSURANCE....................60
14.1 Financing 60
14.2 Insurance....................60
14.3 Compensation 60
15 SERIOUS BREACHES....................61

©Oxford University

16 PUBLICATION POLICY 62

17 REFERENCES 63

©Oxford University

1 SYNOPSIS

**Trial Title**	A phase I clinical trial to determine the safety and immunogenicity of the candidate rabies vaccine ChAdOx2 RabG in UK healthy adult volunteers
**Trial Centre**	Centre for Clinical Vaccinology & Tropical Medicine, University of Oxford, Churchill Hospital, Old Road, Headington, Oxford, OX3 7LE
**Trial Identifier**	RAB001
**Clinical phase**	I
**Study Design**	Open–label, non-randomised, dose escalation, first-in-human, single centre, phase I clinical trial
**Population**	Healthy adults aged 18 – 65 years
**Planned Sample Size**	12-18 volunteers

Group	Dose of ChAdOx2 RabG
Group 1 (n=3)	5×10^{9} vp
Group 2 (n=3)	2.5×10^{10} vp
Group 3 (n=6)	5×10^{10} vp

**Follow-up duration**	8 weeks post-ChAdOx2 RabG administration for each participant. Participants will be given the option to return 52 weeks post-ChAdOx2 RabG administration to receive three doses of inactivated rabies vaccine (IRV) over 3 weeks. This would give a maximum follow-up of 55 weeks.
**Planned Trial Period**	October 2019 to June 2021
**Primary Objective**	To assess the safety profile of ChAdOx2 RabG in healthy adult volunteers

©Oxford University

**Secondary Objective**	To assess the immunogenicity of ChAdOx2 RabG in healthy adult volunteers
**Investigational Products**	A. ChAdOx2 RabG, a replication-deficient simian adenoviral vector expressing the glycoprotein of the rabies virus.<br>B. Inactivated rabies vaccine (IRV). The IRV used will be one of the following, depending on availability;<br>1. Rabies Vaccine BP, manufactured by Sanofi Pasteur and licensed for use in the UK.<br>2. Rabipur, manufactured by GSK Vaccines and licensed for use in the UK.<br>3. Verorab, manufactured by Sanofi Pasteur, licensed for use in several EU countries, and imported to the UK with the agreement of the Department of Health and MHRA for unlicensed use when other products are unavailable.
**Dose per Administration**	ChAdOx2 RabG 5 × 10^{9} vp<br>ChAdOx2 RabG 2.5 × 10^{10} vp<br>ChAdOx2 RabG 5 × 10^{10} vp<br>IRV ≥2.5 international units
**Form**	Liquid
**Route**	Intramuscularly (IM) into the deltoid region of the arm

©Oxford University

2 ABBREVIATIONS

AdC	Chimpanzee adenovirus
AdC68	Chimpanzee Adenovirus serotype 68
AdHu	Human adenovirus
AdHu5	Human adenovirus serotype 5
AE	Adverse event
AR	Adverse reaction
BAC	Bacterial artificial chromosome
CBF	Clinical Biomanufacturing Facility
CCVTM	Centre for Clinical Vaccinology and Tropical Medicine
ChAd63	Chimpanzee Adenovirus serotype 63
ChAdOx1	Chimpanzee Adenovirus Ox1
ChAdOx2	Chimpanzee Adenovirus Ox2
ChAdOx2 RabG	Recombinant Chimpanzee Adenovirus Ox2 with rabies glycoprotein
CI	Chief Investigator
CMV	Human cytomegalovirus
CRF	Case Report Form
CTRG	Clinical Trials Research Governance
DALYs	Disability-adjusted life years
DSUR	Development Safety Update Report
EBV	Epstein Barr virus
ELISA	Enzyme linked immunosorbent assay
ELISpot	Enzyme linked immunospot assay
GCP	Good Clinical Practice
GMO	Genetically modified organism
GMP	Good Manufacturing Practice
GP	General Practitioner
HBsAg	Hepatitis B surface antigen
HCG	Human Chorionic Gonadotrophin
HCV	Hepatitis C virus
HEK	Human embryonic kidney
HIV	Human immunodeficiency virus
HLA	Human leukocyte antigen
IB	Investigators Brochure
ICH	International Conference on Harmonisation
ID	Intradermal
IFN	Interferon
IM	Intramuscular/intramuscularly
IMP	Investigational medicinal product

©Oxford University

IRV	Inactivated rabies vaccine
ISF	Investigator Site File
IU	Infectious units
LSC	Local Safety Committee
MHRA	Medicines and Healthcare products Regulatory Agency
MVA	Modified Vaccinia Virus Ankara
PCR	Polymerase Chain Reaction
PEP	Post-exposure prophylaxis
PrEP	Pre-exposure prophylaxis
PIS	Participant information sheet
QP	Qualified Person
RabG	Rabies glycoprotein
RABV	Rabies virus
REC	Research Ethics Committee
RIG	Rabies immunoglobulin
RSV	Respiratory syncytial virus
SAE	Serious Adverse Event
SAR	Serious Adverse Reaction
SFC	Spot forming cells
SmPC	Summary of Product Characteristics
SOP	Standard Operating Procedure
SUSAR	Suspected Unexpected Serious Adverse Reaction
TMF	Trial Master File
VNA	Virus neutralising assay
vp	Viral particles
WHO	World Health Organisation

©Oxford University

3 BACKGROUND & RATIONALE

3.1 Rabies

Rabies is a neglected tropical disease caused by an enveloped, single-stranded RNA virus of the *Rhabdoviridae* family. The rabies virus (RABV) genome consists of five genes encoding glycoprotein, matrix protein, nucleoprotein, phosphoprotein, and the viral RNA polymerase (1, 2).

RABV has a wide host-range and spreads via the saliva of an infected animal coming into contact with a wound or mucosal surface. The virus spreads primarily though the peripheral nervous system to the central nervous system, where the most significant symptoms leading to death occur. The incubation period ranges from several days to several years in humans, with 2-3 months being most common. Without vaccination, infection results in acute encephalitis and death, although treatment with post-exposure prophylaxis (PEP) is highly effective if it occurs soon after exposure (2, 3).

3.2 Impact of rabies

The belief that rabies is a bygone disease for which an adequate vaccine exists is incorrect. The annual, global human impact of rabies is approximately 59,000 deaths, the loss of 3.7 million disability-adjusted life years (DALYs) and US$ 8.6 billion in economic costs. The greatest burden of impact is felt in Asia and Africa, where 95% of deaths occur (2, 4).

Around 99% of human exposures to rabies is through dog bites, although transmission via bats is the primary mode of transmission in the Americas. Following potential exposure, the cost of PEP treatment represents 31 days' wages for the average Asian and 51 days wages for the average African (2). Even when costs are not prohibitive to individuals, PEP is often unavailable in local clinics when needed.

3.3 Current rabies control and vaccination

Rabies control is multifaceted, including pre-exposure prophylaxis (PrEP) and PEP in humans, and vaccination of animal reservoirs.

The first rabies vaccine was developed in the 19th century by Pasteur, making it the second human disease for which a vaccine was successfully developed. Recommended current vaccines are produced using cell culture systems and are used for both PrEP and PEP. In some countries vaccines are still produced using mammalian nervous system tissue, which the WHO strongly recommends against (2).

The WHO released a new position paper on the use of rabies vaccines in April 2018 (5, 6). This included the recommendation of the greater use of intradermal (ID) over intramuscular (IM) administration and new regimens for PrEP and PEP. These changes are primarily aimed at decreasing costs and improving access and adherence, while maintaining comparable levels of protection.

©Oxford University

3.3.1 Pre-exposure prophylaxis (PrEP)

PrEP usually involves 3 doses of vaccine spread over several weeks. The new WHO guidelines suggest 2 doses of vaccine spread over one week is sufficient for protection (6). The UK Department of Health Green Book still recommends the 3 dose regimen for UK vaccinees (7). Experience with single-visit regimes with existing licensed vaccines is limited but available evidence suggests that a substantial proportion of recipients of such regimes fail to sero-convert and antibody titer maintenance is relatively poor (8-10).

3.3.2 Post-exposure prophylaxis (PEP)

For individual at high risk of rabies exposure that have not been previously vaccinated, PEP usually involves a combination of multiple doses of vaccine plus rabies immunoglobulin (RIG) spread over 4-5 visits and 3-4 weeks. The new WHO guidelines suggest 3-4 doses of vaccine spread over 1-3 weeks, with RIG only for the most serious level of exposure (6).

For individuals at high risk of rabies exposure who have been previously vaccinated, PEP involves 2 doses of vaccine up to 3 days apart, and no RIG (6).

3.3.3 Animal vaccination

The major vectors are dogs in Asia and Africa. Unfortunately, many parenteral dog vaccination campaigns fail to reach adequate levels of coverage (11); oral vaccines are less effective due to canine feeding habits/ anatomy. Dog vaccination has been endorsed by the Global Alliance for Rabies Control and enshrined in the Zero by Thirty strategy as the preferred route to reduce human rabies mortality (12, 13). However, funding for this approach remains poor and many regions lack – and will likely continue to lack – the animal health infrastructure to implement this.

3.4 The need for a new vaccine

These existing control measures are inadequate: current human vaccines are too expensive for mass use, and canine vaccination is challenging in many settings.

Existing inactivated-virus human rabies vaccines require expensive manufacturing processes, repeated dosing and, with one exception, cold-chain storage. They are not cost-effective for PrEP in low-income settings (14). PEP is prohibitively expensive, representing 51 day's wages for the average African, and not promptly available in areas where most cases occur (2, 4, 14).

Despite the spread of canine vaccination as a method of control, with significant success in certain areas, global incidence is stable and, in some areas, rising. In many areas there is a lack of animal health infrastructure to achieve the necessary >70% coverage in dog populations that are highly mobile and turn over every 2 years. Rural settings, where dog populations are more dispersed, are particularly challenging. Yet in many such settings high

©Oxford University

rates of routine paediatric immunisation have been achieved, e.g. against measles, suggesting routine population-wide PrEP as a pathway to effectively reduce human rabies mortality in areas which struggle to implement canine vaccination.

A low cost, single dose, efficacious human rabies vaccine would thus be a major advance for rabies control. Rabies elimination would also require control in dogs and other reservoirs, but a new human vaccine has the potential to have a major impact on global mortality and economic costs arising from rabies.

3.5 Rabies glycoprotein as a vaccine antigen

Rabies glycoprotein (RabG) is the key surface antigen against which protective neutralising virus antibodies are induced by currently available vaccines (15). Thus, millions of individuals have previously been safely administered this antigen. Vaccination with RabG via adenovirus vectors has been shown to induce RabG antibody titers comparable to currently available vaccines in animal models, and to protect non-human primates from rabies challenge (15, 16). ChAdOx2 RabG contains the full length RabG sequence.

3.6 Adenovirus-vectored vaccines

Adenoviruses are attractive vectors for human vaccination. They possess a stable genome so that inserts of foreign genes intended for *in vivo* expression are maintained during repeated culture. They can enter a wide variety of cell types and their genetic material remains extra-chromosomal, meaning there is no potential for insertional mutagenesis in host cells. Replication incompetent adenovirus vectors have been engineered through the deletion of the E1 locus, ensuring there is no replication within vaccine recipients' cells. These viruses can still be propagated with high yields *in vitro* using cell lines expressing the E1 locus, such as human embryonic kidney 293 cells (HEK 293 cells) (17).

Previous mass vaccination campaigns in over 2 million adult US military personnel using orally administered live human adenovirus (AdHu) serotype 4 and 7 have shown good safety and efficacy data (18). AdHus are under development as vectors for malaria, HIV and hepatitis C vaccines, amongst others. They have been used extensively in human trials with a consistently excellent safety profile.

A limiting factor to widespread use of human adenovirus as vaccine vectors has been the level of pre-existing immunity, with seroprevalence of up to 90% reported in sub-Saharan Africa (19, 20). This limits the immunogenicity of the AdHu vectored vaccines, with several animal models showing prior exposure to AdHu attenuates responses to subsequent challenge with AdHu vectored vaccines but not Chimpanzee adenovirus (AdC) vectored vaccines, including against rabies glycoprotein (21-23). The ideal adenovirus vector would be replication deficient, not cause human disease, produce high yields during manufacture, and be highly immunogenic. AdCs that have these characteristics are increasingly accepted as widely applicable vectors for human use (24, 25).

©Oxford University

Key to their applicability as vaccine vectors is that AdCs exhibit hexon structures that show enough homology to AdHu hexon to enable effective infection of human cells, while circumventing pre-existing immunity to the most common AdHu serotypes (24, 26). Several trials have now shown excellent immunogenicity in a variety of unselected human populations, including children in low-income settings (27-29). Moreover, the vectors have an excellent track record of safety and tolerability (drawn from experience in >1000 vaccinees with Oxford-developed vaccines alone). Industrial confidence in the platform is demonstrated by heavy investment by both Johnson & Johnson and GSK: both have purchased companies developing simian adenovirus vaccines, are involved in Phase II/III trials of adenovirus-based Ebola vaccines, and have invested in adenovirus manufacturing capabilities. Chimpanzee adenoviral vectors can be manufactured cost-effectively (30) and are now in clinical development as possible vaccines against malaria, HIV, tuberculosis, influenza, hepatitis C, RSV, cancer and Ebola.

3.7 ChAdOx2

ChAdOx2 is a replication incompetent adenovirus vector derived from wild type AdC serotype C68 (AdC68) and ChAdOx1, a viral vector previously developed in the Jenner Institute. ChAdOx1 is described by Dicks *et al.* (31) and has had a good safety profile in numerous clinical trials. ChAdOx1 was constructed in a bacterial artificial chromosome (BAC) to facilitate genetic manipulation of genomic clones with improved stability and flexibility. Cellular immunogenicity of ChAdOx1 was comparable to that of other species E derived chimpanzee adenovirus vectors including ChAd63, the first simian adenovirus vector to enter clinical trials in humans. ChAdOx2 was then produced in the same manner, starting from the replication-competent AdC68 with further modification to the E4 region to increase yields during manufacture (24). As ChAdOx2 is replication incompetent, E4 is not expressed in vaccine recipients. This modified E4 region was derived from AdC63 and ADHu5, both of which have a good safety profile in clinical trials.

Serotype Y25 (from which ChAdOx1 is derived), AdC68 (also known as SAdV25, and from which ChAdOx2 is derived), and ChAd63 are all species E adenoviruses and have a close phylogenetic relationship when nucleotide sequences of the hexon and fibre proteins are analysed (Figure 1).

©Oxford University

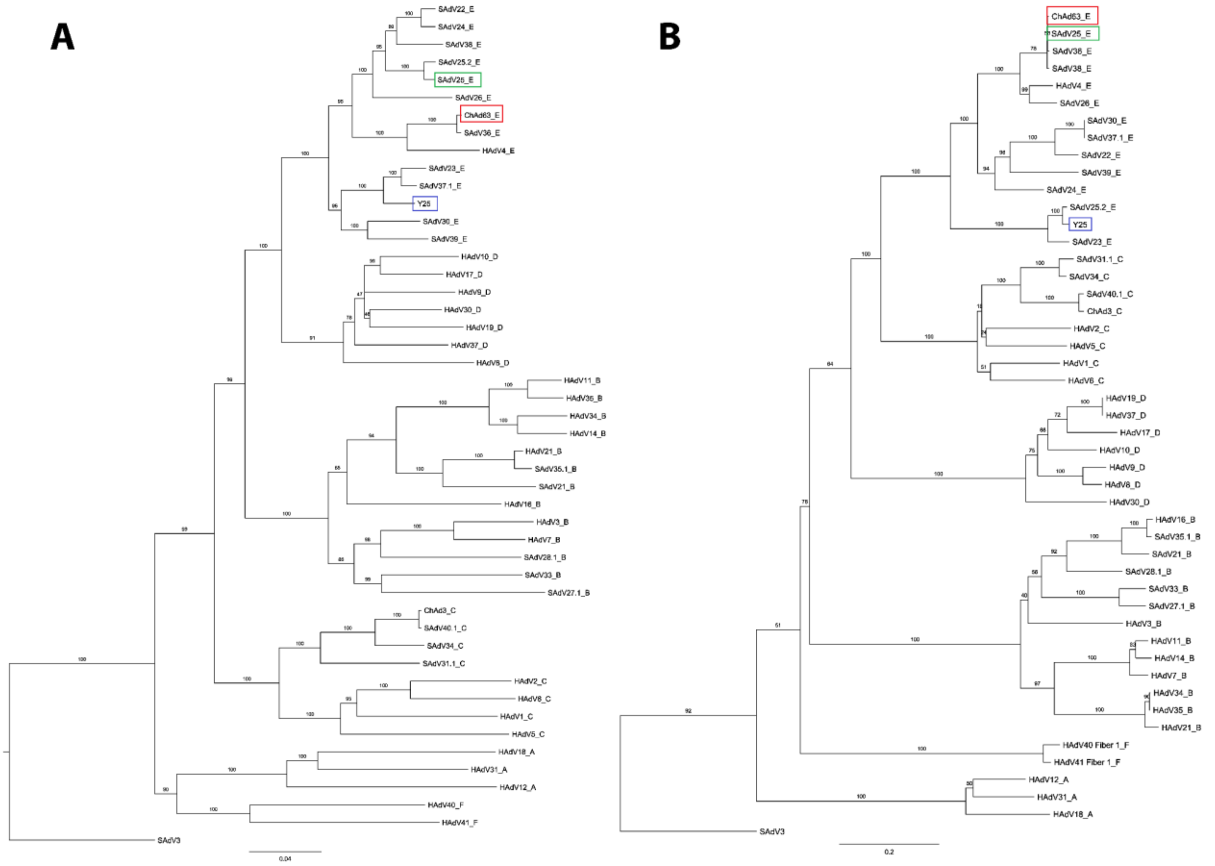

Figure 1. Phylogenetic trees based on alignment of nucleotide sequences of (A) hexon and (B) the fibre protein of different human and chimpanzee adenovirus serotypes. AdC68 (SAdV25; green), Y25 (blue) and ChAd63 (red) are highlighted. Modified from (31).

3.8 Development of ChAdOx2 vaccine vector

To generate a molecular clone of the AdC68 genome, a BAC gap repair vector was constructed containing PCR-amplified regions of homology to the left and right flanks of the viral genome as described in Chartier *et al* (32). An extra homology flank downstream of the adenovirus E1 region was included to enable deletion of E1 and placement of a unique restriction site at the E1 locus, concomitant with genomic insertion into the BAC. The E1 region is essential for viral replication, hence the ability to delete E1 at this stage renders the new vector immediately replication incompetent. Replication incompetent (E1-deleted) clones were successfully identified by PCR screening and transfection into E1 complementing HEK293 cells confirmed the ability of all candidate clones of the new vector to generate infectious virions. The non-essential E3 region was subsequently deleted to increase the insertion capacity of the vector.

Proteins encoded by the E4 region interact with E1 proteins during viral replication, and the imperfect interaction between the gene products of the AdHu5 E1 gene produced by HEK293 cells and simian E4 gene products has been found to result in impaired viral replication in this cell line, and consequently lower virus yields (26, 31, 33). In ChAdOx1, Ad5 E4Orf4,6/7 have been inserted to replace the homologous simian virus coding sequence, resulting in improved viral replication during vaccine production (31). Since no replication of the virus takes place

©Oxford University

after immunization, this replacement has no effect on immunogenicity of the viral vector. In the construction of ChAdOx2, the whole of the native AdC68 E4 region was replaced with the equivalent regions from ChAdOx1.

3.9 Development of ChAdOx2 RabG

ChAdOx2 RabG encodes the rabies glycoprotein. A genomic clone of ChAdOx2 RabG was prepared by Gateway® recombination between an entry plasmid containing the native coding sequence for glycoprotein from the ERA strain rabies virus under the transcriptional control of an Intron-A containing human cytomegalovirus immediate-early promoter, and the E1-and E3-deleted ChAdOx2 destination vector.

3.10 Preclinical studies

Studies have shown ChAdOx2 RabG to be immunogenic, inducing both antibody (15) and T cell responses in mice. Full details are available in the current version of the ChAdOx2 RabG Investigator's Brochure (IB).

In a non-human primate trial, an AdC68 vectored RabG vaccine outperformed the current human rabies vaccine: a single dose protected 100% of animals challenged 22 months after vaccination (16).

3.11 Previous clinical experience

This will be the first-in-human clinical trial of ChAdOx2 RabG. The gene product of the antigenic insert, RabG, is present in several currently licenced rabies vaccines, and thus has been administered successfully to millions of individuals.

Another ChAdOx2 vectored vaccine expressing a different insert has been administered to 12 healthy volunteers taking part in a clinical trial in the UK conducted by the University of Oxford. This vaccine was successfully administered at the same doses proposed in the current study, and there were no serious adverse events (SAEs) associated with the vaccine reported to date. The number of participants and doses are outlined in Table 1.

©Oxford University

Table 1. University of Oxford clinical experience with ChAdOx2 viral vectored vaccines.

**Country**	**Trial**	**Vaccine**	**Age**	**Route**	**Dose**	**Number of Volunteers (Received ChAdOx2 up to 1 May 2019)**
UK	HAV001	ChAdOx2 HAV	18-50	IM	5×10^{9} vp	3
					2.5×10^{10} vp	3
					5×10^{10} vp	6

Chimpanzee adenovirus vaccine vectors have been safely administered to over 1000 people, targeting a wide range of pathogens (and also cancer). ChAdOx1 is a chimpanzee adenovirus vaccine vector system that has been administered to over 200 healthy volunteers taking part in clinical trials in the UK conducted by the University of Oxford. The vaccine studies, doses, and number of participants are outlined in Table 2. ChAdOx1 and ChAdOx2 are derived from closely related species E chimpanzee adenoviruses, modified in similar ways and sharing a region of non-structural gene sequence (see section 3.7 and Figure 1), making ChAdOx1 safety data relevant to consideration of ChAdOx2.

ChAd63 is another chimpanzee adenovirus vaccine vector system successfully and safely administered to human participants in a range of clinical trials (34). ChAd63 is also a species E chimpanzee adenovirus closely related to the virus from which ChAdOx2 RabG is derived (see section 3.7 and Figure 1).

©Oxford University

Table 2. University of Oxford clinical experience with ChAdOx1 viral vectored vaccines.

Country	Trial	Vaccine	Age	Route	Dose	Number of Volunteers (Received ChAdOx1 up to 1 May 2019)
UK	FLU004	ChAdOx1 NP+M1	18-50	IM	5×10^8 vp	3
					5×10^9 vp	3
					2.5×10^{10} vp	3
					5×10^{10} vp	6
UK	FLU005	ChAdOx1 NP+M1<br>MVA NP+M1 (week 8)	18-50	IM	2.5×10^{10} vp	12
		ChAdOx1 NP+M1<br>MVA NP+M1 (week 52)	18-50	IM	2.5×10^{10} vp	12
		MVA NP+M1<br>ChAdOx1 NP+M1 (week 8)	18-50	IM	2.5×10^{10} vp	12
		MVA NP+M1<br>ChAdOx1 NP+M1 (week 52)	18-50	IM	2.5×10^{10} vp	9
		ChAdOx1 NP+M1	>50	IM	2.5×10^{10} vp	12
		ChAdOx1 NP+M1<br>MVA NP+M1 (week 8)	>50	IM	2.5×10^{10} vp	12
UK	TB034	ChAdOx1 85A	18-50	IM	5×10^9 vp	6
					2.5×10^{10} vp	12
		ChAdOx1 85A<br>MVA85A (week 8)	18-50	IM	2.5×10^{10} vp	12
UK	VANCE01	ChAdOx1.5T4<br>MVA.5T4	18 – 75	IM	2.5×10^{10} vp	34
UK	VAC067	ChAdOx1 LS2	18-45	IM	5×10^9 vp	3
					2.5×10^{10} vp	10
UK	MERS001	ChAdOx1 MERS	18-50	IM	5×10^9 vp	6
					2.5×10^{10} vp	9
					5×10^{10} vp	9
UK	CHIK001	ChAdOx1 Chik	18-50	IM	5×10^9 vp	6
					2.5×10^{10} vp	9
					5×10^{10} vp	9
UK	VAMBOX	ChAdOx1 MenB.1	18-50	IM	$2.5 x 10^{10}$ vp	3
					$5 x 10^{10}$ vp	24

©Oxford University

3.12 Rationale

Rabies causes significant mortality and hardship globally, particularly in low and middle income countries where it is endemic. Although both pre- and post-exposure prophylaxis is available, costs for both are too high, which coupled with the need for multiple visits, prevents their full potential being realised. Pre-exposure prophylaxis is too expensive for large-scale vaccination campaigns that have been used to combat other diseases like measles. Post-exposure prophylaxis carries significant out of pocket expenses for many people that lead to it being unaffordable or causing financial stress. ChAdOx2 RabG has the potential to lower costs and make mass vaccination campaigns realistic.

Chimpanzee adenovirus vaccine vectors have been safely administered to thousands of people using a wide range of infectious disease targets. ChAdOx2 viral vectored vaccines have shown to be both safe and immunogenic in a previous clinical trial (HAV 001) (35). Single-dose immunisation with the ChAdOx2 RabG vaccine has shown to elicit high levels of neutralising antibody in animal models. This study will provide valuable data on safety, immunology, and longevity or responses against RabG following vaccination with ChAdOx2 RabG.

3.13 Vaccine development strategy

The data from this first in human trial of ChAdOx2 RabG will be used to support further Phase I trials in the target population. If this study shows a good safety profile, a Phase Ib trial will be carried out in a rabies endemic region, confirming the safety of the preferred dose suggested by this study in adult volunteers. This will be followed by age de-escalation and dose-escalation in the ultimate target group, children aged 1-6 years old.

3.14 Rationale for optional extended follow-up including immunisation with existing rabies vaccine

During the optional period of follow-up in this study, participants will receive a currently available inactivated rabies vaccine (IRV) according to the recommended UK schedule (see section 3.3.1). The scientific goal of the administration of IRV is to measure the memory immune response that was induced by ChAdOx2 RabG following re-exposure to the rabies glycoprotein: by administering IRV, we will effectively be measuring the secondary or recall response to ChAdOx2 RabG. The strength and kinetics of such recall responses may ultimately be a key factor in determining the efficacy of ChAdOx2 RabG in future clinical trials.

The strength of the immune response following each of the first and second doses of IRV is of particular interest. This is because current PEP guidelines suggest individuals previously vaccinated against rabies but potentially exposed to infection should receive ‘abbreviated PEP’ (2 doses of IRV to boost their immune response see section 3.3.2): in other words, follow-up of the immune response after two doses of IRV will model what would be expected if an

©Oxford University

individual who had received ChAdOx2 RabG as pre-exposure prophylaxis was subsequently bitten by a suspected rabid animal and received abbreviated PEP.

Although administration of two doses of IRV (and subsequent follow-up) would be sufficient to model abbreviated PEP and to meet the scientific goals of the trial, we have chosen to administer *three* doses because this is the currently recommended schedule for *PrEP* in the UK (see section 3.3.1). We feel it is in the volunteers' best interests to have obtained a full course of PrEP according to the recommendations, independent of the efficacy or otherwise of the experimental ChAdOx2 RabG vaccine. We do not intend to follow up the volunteers after the third dose for the following reasons:

1. We see no scientific value in sampling at later timepoints. After three doses IRV, all volunteers would be expected to sero-convert regardless of the performance of the ChAdOx2 RabG.
2. We see no participant safety need for further follow up. The SmPCs for IRVs do not recommend any routine follow-up. These timepoints will be around a year after receipt of ChAdOx2 RabG and so we do not believe that there is any plausible mechanism by which having received ChAdOx2 RabG could increase the very low risk associated with IRV administration.

In this study, we have listed three different IRVs which may be used during the optional follow-up period. This is due to global shortages of IRV supply, which impact availability in the UK. It is not possible 12 months ahead of their use to accurately predict which IRV/s will be available at the time for use during this follow-up period, and thus which one of the three listed IRVs will be used.

3.15 Rationale for optional saliva sample collection and analysis

The study provides a valuable opportunity to collect serial samples from a group of healthy volunteers who will be returning to the clinic repeatedly, and so we plan to request saliva samples from the volunteers, for purposes unconnected to the ChAdOx2 RabG vaccine. Provision of a saliva sample is quick, painless, risk-free and therefore of no additional burden to the volunteers, but this will nonetheless be optional.

The Chief Investigator's research group is also interested in the development of vaccines against two other viruses, Epstein Barr Virus (EBV) and Human Cytomegalovirus (CMV). Once an individual is infected with either of these viruses, it persists for life and is frequently shed in the individual's saliva (the main route of transmission). These viruses are very common in healthy individuals in the UK: more than 90% are infected with EBV and around 50% with CMV (36), and as such it is likely that several of the volunteers in this study will be infected with each virus. They very rarely cause serious problems but can do so in certain contexts (for example if CMV is acquired for the first time during pregnancy).

©Oxford University

At present there is no effective vaccine against either virus. One challenge in developing a vaccine is the question of how to assess its efficacy in humans within a trial of realistic size and duration.

We hypothesize that it may be possible, in future, to test candidate vaccines against these viruses by testing the vaccine's ability to induce an immune response which suppresses viral replication in volunteers who are *already infected*, and hence suppresses salivary shedding of the virus. In other words, salivary shedding would be used as a biomarker of vaccine efficacy. There is precedent for a vaccine being effective in suppressing viral replication in people already infected with a related virus (the licensed vaccines against shingles, caused by re-activation of varicella zoster virus).

In order to assess the possible value of salivary virus shedding as a biomarker in future studies, we need to understand the pattern and variability of shedding over time in healthy volunteers receiving a vaccine unrelated to EBV or CMV (we are also performing a separate pilot study in healthy volunteers not receiving any vaccine). The samples will be used for studies of the shed virus (principally, quantitative PCR). The results of such studies would have no implications for the volunteers' health.

Participants may choose to take part in either one, both or neither of the optional parts of this study.

©Oxford University

4 OBJECTIVES AND ENDPOINTS

4.1 Primary Objective

To assess the safety and tolerability of ChAdOx2 RabG in healthy volunteers.

4.1.1 Primary Outcome Measures

The specific endpoints for safety and reactogenicity will be actively and passively collected data on adverse events.

The following parameters will be assessed for all study groups.

- Occurrence of solicited local reactogenicity signs and symptoms for 7 days following the vaccination.
- Occurrence of solicited systemic reactogenicity signs and symptoms for 7 days following the vaccination.
- Occurrence of unsolicited adverse events for 28 days following the vaccination.
- Change from baseline for safety laboratory measures.
- Occurrence of serious adverse events during the whole study duration.

SAEs will be collected throughout the study, including at later timepoints for those volunteers participating in the optional extended follow up.

4.2 Secondary Objective

To assess the immunogenicity of ChAdOx2 RabG in healthy adult volunteers.

4.2.1 Secondary Outcome Measures

Immunogenicity of the ChAdOx2 RabG vaccine will be assessed by rapid fluorescent focus inhibition test (RFFIT) of virus neutralising antibody (37).

Measurements of immunogenicity may include assays performed in laboratories outside Europe. This would involve transfer of serum, plasma and/or peripheral blood mononuclear cells (PBMC), but samples would be de-identified and this will be explicitly covered by informed consent.

4.3 Additional (exploratory) Objectives

In the optional extended follow-up study: To study immunological memory induced by ChAdOx2 RabG, by means of measuring the 'recall' response upon re-encounter of rabies glycoprotein, virus neutralizing antibody will be measured before and in the course of immunisation with IRVs.

In samples from volunteers consenting to the optional saliva collection, the levels and timecourse of EBV and CMV shedding will be measured by quantitative PCR.

©Oxford University

Other exploratory immunological assays may be carried out at the Jenner Institute or in collaboration with other specialist laboratories. Such studies would not include any assay with potential health implications for the volunteers.

Explorative immunology may including laboratories outside of Europe. This would involve transfer of serum, plasma and/or peripheral blood mononuclear cells (PBMC), but samples would be de-identified and this will be explicitly covered by informed consent.

©Oxford University

5 STUDY OVERVIEW

This is a first-in-human, open-label, dose escalation, phase I clinical trial to assess the safety and immunogenicity of the candidate ChAdOx2 RabG vaccine in healthy UK volunteers aged 18-65. The vaccine will be administered intramuscularly (IM).

Volunteers will be recruited and vaccinated at the Centre for Clinical Vaccinology and Tropical Medicine (CCVTM), Oxford. There will be 3 study groups and it is anticipated that a total of 12 volunteers will be enrolled. Staggered enrolment will apply between study groups and for the first three volunteers within each group. Volunteers will be first recruited into Group 1 and subsequently into Groups 2 and 3 following interim clinical safety reviews (see section 5.3). Volunteers will be sequentially allocated to a study group by selecting eligible volunteers for enrolment following screening. Sequential allocation will occur based on order in which volunteers are enrolled.

The study includes an optional extended follow-up period, lasting one month and starting one year after vaccination. Volunteers will receive a complete pre-exposure prophylactic course of an existing rabies vaccine, allowing study of the immunological memory (recall response) induced by ChAdOx2 RabG.

A second optional element of the study is the collection of saliva samples at each visit for the study of shedding of EBV and CMV.

5.1 Rationale for Selected Doses

Doses to be administered in this trial have been selected on the basis of clinical experience with the ChAdOx2 adenovirus vector expressing a different insert, and similar adenovirus vectored vaccines (e.g. ChAdOx1 and ChAd63).

ChAdOx2 HAV has been safely administered at doses ranging from 5×10^9 to 5×10^{10} vp following a similar dose-escalation approach (35).

ChAdOx1 NP+M1 has been safely administered at doses ranging from 5×10^8 to 5×10^{10} vp with an optimal dose of 2.5×10^{10} vp, balancing immunogenicity and reactogenicity. This dose has subsequently been given to over 100 volunteers in numerous larger phase I studies at the Jenner Institute (FLU005, TB034 VANCE01 and VAC067) and ChAdOx1 vectored vaccines have thus far been very well tolerated. The vast majority of adverse events (AEs) have been mild-moderate and there have been no severe adverse reactions (SARs) until this date.

ChAd63 has been safely administered at doses up to 2×10^{11} vp with an optimal dose of 5×10^{10} vp, balancing immunogenicity and reactogenicity.

For safety reasons, the first dose of ChAdOx2 RabG proposed in this study (5×10^9 vp) is therefore at least 10 fold less than what is expected to be tolerated (5×10^{10} vp). Doses will be gradually increased aiming to identify an optimal dose of ChAdOx2 RabG considering the tolerability, reactogenicity and immunogenicity profiles.

©Oxford University

5.2 Study Groups

Table 3. Study groups.

Group	Single Dose ChAdOx2 RabG	Route
Group 1 (n=3)	5×10^{9} vp	IM
Group 2 (n=3)	2.5×10^{10} vp	IM
Group 3 (n=6)	5×10^{10} vp	IM

5.3 Vaccination and safety reviews

The first volunteer in Group 1 will be vaccinated alone and safety data reviewed 48 hours later. The Chief Investigator (CI) and the Chair of the local safety committee (LSC) will decide whether to proceed after this safety review of the first volunteer. If there are no safety concerns, another two Group 1 volunteers may be vaccinated at least one hour apart. An independent safety review will be conducted by the CI and Chair of the LSC after vaccination of the 3 volunteers in Group 1. This review will include the results of safety blood tests at day 7 post vaccination and an assessment of the profile of the adverse events reported. The CI and Chair of the LSC will decide whether to proceed with vaccination at the next highest dose (Group 2) following safety review of the previously vaccinated volunteers.

The same procedures will apply for Group 2.

The same procedures will apply for Group 3, except that the independent safety review of the first 3 volunteers in Group 3 will inform the CI and Chair of the LSC decision whether to proceed with vaccination of the remaining 3 volunteers in Group 3.

Should the CI and Chair of the LSC have safety concerns during any safety review, holding rules may be activated (see section 9.9) or they may decide to vaccinate more volunteers in Groups 1 or 2, up to a maximum total of 6 volunteers per group.

5.4 Duration of study

The total duration of the study will be 55 weeks from the day of enrolment for each volunteer. Follow-up for all volunteers will be 8 weeks. Volunteers will be given the option to return 52 weeks post-ChAdOx2 RabG administration to receive three doses of IRV over 3 weeks.

5.5 Definition of Start and End of Trial

The start of the trial is defined as the date of the first vaccination of the first volunteer. The end of the trial is the date of the last visit of the last volunteer.

©Oxford University

5.6 Potential Risks for volunteers

The potential risk to participants is considered as low. The potential risks are those associated with phlebotomy and vaccination. In general, recombinant adenoviral vectors are safe. Similar vaccines encoding different antigens have been given to several thousand volunteers (including children) with a good safety profile.

Phlebotomy:

The maximum volume of blood drawn over the study period (approximately 484 mL) should not compromise these otherwise healthy volunteers. There may be minor bruising, local tenderness or pre-syncopal symptoms associated with venepuncture, which will not be documented as AEs if they occur.

Vaccination:

Potential foreseeable risks from vaccination include local effects such as pain, redness, warmth, swelling, tenderness or itching. Systemic reactions that could potentially occur following immunisation with a recombinant adenovirus vaccine include a flu-like illness with feverishness, fatigue, malaise, arthralgia, myalgia and headache.

As with any vaccine, Guillain-Barré syndrome or immune-mediated reactions that can lead to organ damage may occur, but this should be extremely rare. Serious allergic reactions including anaphylaxis could also occur and for this reason volunteers will be vaccinated in a clinical area where Advanced Life Support trained physicians, equipment and drugs are immediately available for the management of any serious adverse reactions (SAR).

5.7 Known Potential Benefits

Volunteers that choose to return for the second follow-up period will benefit directly from participation in this study through the receipt of IRV, which is known to induce protective immunity against rabies. It is also hoped that the information gained from this study will contribute to the development of a cheaper and more effective rabies vaccine regime, providing benefits to broader society. The only other benefit for participants would be limited information about their general health status.

©Oxford University

6 RECRUITMENT AND WITHDRAWAL OF TRIAL VOLUNTEERS

6.1 Volunteers

Volunteers may be recruited by use of an advertisement and/or registration form formally approved by the ethics committee(s) and distributed or posted in the following places:

- In public places, including buses and trains, with the agreement of the owner/proprietor.
- In newspapers or other literature for circulation.
- On radio via announcements.
- On a website or social media site operated by our group or with the agreement of the owner or operator (including on-line recruitment through our web-site).
- By e-mail distribution to a group or list only with the express agreement of the network administrator or with equivalent authorisation.
- By email distribution to individuals who have already expressed an interest in taking part in any clinical trial at the Oxford Vaccine Centre.
- On stalls or stands at exhibitions or fairs.
- Via presentations (e.g. presentations at lectures or invited seminars).
- Direct mail-out: This will involve obtaining names and addresses of adults via the most recent Electoral Roll. The contact details of individuals who have indicated that they do not wish to receive postal mail-shots would be removed prior to the investigators being given this information. The company providing this service is registered under the Data Protection Act 2018. Investigators would not be given dates of birth or ages of individuals, but the list supplied would only contain names of those aged between 18-65 years (as per the inclusion criteria).
- Oxford Vaccine Centre databases: We may contact individuals from databases of groups within the CCVTM (including the Oxford Vaccine Centre database) of previous trial participants who have expressed an interest in receiving information about all future studies for which they may be eligible.

6.2 Informed consent

All volunteers will sign and date the informed consent form before any study specific procedures are performed. The information sheet will be made available to the volunteer at least 24 hours prior to the screening visit. At the screening visit, the volunteer will be fully informed of all aspects of the trial, the potential risks and their obligations. The following general principles will be emphasised:

- Participation in the study is entirely voluntary
- Refusal to participate involves no penalty or loss of medical benefits
- The volunteer may withdraw from the study at any time
- The volunteer is free to ask questions at any time to allow him or her to understand the purpose of the study and the procedures involved

©Oxford University

- The study involves research into an investigational vaccine
- The only benefit from participating is receipt of the licenced rabies vaccine
- The volunteer's General Practitioner (GP) will be contacted to corroborate their medical history
- The volunteer's blood samples taken as part of the study will be stored indefinitely and samples may be sent outside of the UK and Europe to laboratories in collaboration with the University of Oxford. These will be de-identified.

Volunteers will be free to opt-in or opt-out of the two optional elements of the study (extended follow-up and IRV vaccination, and saliva sampling), by providing additional informed consent for these elements, independent of consent for the core study.

The aims of the study and all tests to be carried out will be explained. The volunteer will be given the opportunity to ask about details of the trial and will then have time to consider whether or not to participate. If they do decide to participate, they will sign and date the consent form, which will then be photocopied. The photocopy will be for the volunteer to take away and keep, and the original will be stored in the case report form (CRF) – this is a paper or electronic document used to collect data relating to a particular volunteer. These forms will also be signed and dated by the Investigator.

6.3 Inclusion and exclusion criteria

This study will be conducted in healthy adults, who meet the following inclusion and exclusion criteria:

6.3.1 Inclusion criteria

The volunteer must satisfy all the following criteria to be eligible for the study:

1. Healthy adults aged 18 to 65 years.
2. Able and willing (in the Investigator's opinion) to comply with all study requirements.
3. Willing to allow the investigators to discuss the volunteer's medical history with their GP.
4. For females only, willingness to practice continuous effective contraception (see section 6.3.4) during the study and a negative pregnancy test on the day(s) of screening and vaccination(s).
5. Agreement to refrain from blood donation during the course of the study.
6. Provide written informed consent.

6.3.2 Exclusion criteria

The volunteer may not enter the study if any of the following apply:

1. Participation in another research study involving receipt of an investigational product in the 30 days preceding enrolment, or planned use during the study period. To be re-confirmed at the enrolment visit.

©Oxford University

2. Prior receipt of an investigational vaccine likely to impact on interpretation of the trial data (e.g. Adenovirus vectored vaccine).
3. Administration of immunoglobulins and/or any blood products within the three months preceding the planned administration of the vaccine candidate.
4. Any confirmed or suspected immunosuppressive or immunodeficient state, including HIV infection; asplenia; recurrent, severe infections and chronic (more than 14 days) immunosuppressant medication within the past 6 months (inhaled and topical steroids are allowed).
5. History of allergic disease or reactions likely to be exacerbated by any component of the vaccine.
6. Any history of hereditary angioedema, acquired angioedema, or idiopathic angioedema.
7. Any history of anaphylaxis in relation to vaccination.
8. Pregnancy, lactation or willingness/intention to become pregnant during the study.
9. History of cancer (except basal cell carcinoma of the skin and cervical carcinoma in situ).
10. History of serious psychiatric condition likely to affect participation in the study.
11. Bleeding disorder (eg. Factor deficiency, coagulopathy or platelet disorder), or prior history of significant bleeding or bruising following IM injections or venepuncture.
12. Any other serious chronic illness requiring hospital specialist supervision.
13. Suspected or known current alcohol abuse as defined by an alcohol intake of greater than 42 units every week.
14. Suspected or known injecting drug abuse in the 5 years preceding enrolment.
15. Detectable circulating hepatitis B surface antigen (HBsAg).
16. Seropositive for hepatitis C virus (antibodies to HCV).
17. Any clinically significant abnormal finding on screening biochemistry or haematology blood tests or urinalysis.
18. Any other significant disease, disorder or finding which may significantly increase the risk to the volunteer because of participation in the study, affect the ability of the volunteer to participate in the study or impair interpretation of the study data.
19. Inability of the study team to contact the volunteer's GP to confirm medical history and safety to participate.
20. Receipt of any prior rabies vaccine, including an incomplete course.
21. Require or will require rabies vaccination during the first 8 weeks of the study period (e.g. through planned travel to high risk enzootic areas of through work which may lead to exposure and for which rabies vaccination is usually required/recommended).

6.3.3 Exclusion criteria for optional follow-up

1. Receiving rabies vaccination following the completion of the first 8 weeks of follow-up but before the optional extended follow-up period will exclude participants from taking part in the optional follow-up period.

©Oxford University

2. History of allergic reactions to amphotericin B, chlortetracycline, neomycin, polymyxin, streptomycin, or to any antibiotics of the same groups will exclude participants from receiving certain IRVs (as per the relevant SmPC) during the optional extended follow-up period, but will not exclude participants from receiving ChAdOx2 RabG.

6.3.4 Effective contraception for female volunteers

Female volunteers are required to use an effective form of contraception during the course of the study (i.e until their last follow up visit). As this is a Phase I, first-in-human, study there is no information about the effect of this vaccine on a foetus. Male participants with female partners of child-bearing potential (i.e. fertile, following menarche and until becoming post-menopausal unless permanently sterile) are not required to use barrier methods for the purposes of contraception whilst taking part in this study as the risk of excretion of the vaccine is negligible.

Acceptable forms of contraception for female volunteers include:

- Established use of oral, injected or implanted hormonal methods of contraception.
- Placement of an intrauterine device (IUD) or intrauterine system (IUS).
- Total abdominal hysterectomy
- Barrier methods of contraception (condom or occlusive cap with spermicide)
- Male sterilisation, if the vasectomised partner is the sole partner for the participant.
- True abstinence: when this is in line with the preferred and usual lifestyle of the participant. Periodic abstinence (e.g., calendar, ovulation, symptothermal, post-ovulation methods), declaration of abstinence for the duration of exposure to IMP, and withdrawal are not acceptable methods of contraception.

6.3.5 Prevention of 'over volunteering'

Volunteers will be excluded from the study if they are concurrently involved in another trial. In order to check this, volunteers will be asked to provide their National Insurance or Passport number (if they are not entitled to a NI number) and will be registered on a national database of participants in clinical trials (*www.tops.org.uk*).

6.3.6 Criteria for postponement of vaccination (Individual holding rules)

The following events constitute contraindications to administration of the vaccine at that point in time; if any one of these events occurs at the time scheduled for vaccination, the participant may be vaccinated at a later date, or withdrawn at the discretion of the Investigator. The participant must be followed until resolution of the event, as with any adverse event.

- Acute disease at the time of vaccination. Acute disease is defined as the presence of a moderate or severe illness with or without fever. All vaccines can be administered

©Oxford University

to persons with a minor illness such as diarrhoea, mild upper respiratory infection with or without low-grade febrile illness, i.e. temperature of ≤37.5°C/99.5°F.

- Temperature of >37.5°C (99.5°F) at the time of vaccination.

6.3.7 Withdrawal of volunteers

In accordance with the principles of the Declaration of Helsinki (2008) and any other applicable regulations, a volunteer has the right to withdraw from the study at any time and for any reason, and is not obliged to give his or her reasons for doing so. The Investigator may withdraw the volunteer at any time in the interests of the volunteer's health and well-being. In addition, the volunteer may withdraw/be withdrawn for any of the following reasons:

- Administrative decision by the Investigator.
- Ineligibility (either arising during the study or retrospectively, having been overlooked at screening).
- Significant protocol deviation.
- Volunteer non-compliance with study requirements.
- An AE, which requires discontinuation of the study involvement or results in inability to continue to comply with study procedures.

The reason for withdrawal will be recorded in the CRF. If withdrawal is due to an AE, appropriate follow-up visits or medical care will be arranged, with the agreement of the volunteer, until the AE has resolved, stabilised or a non-trial related causality has been assigned. Any volunteer who is withdrawn from the study may be replaced, if that is possible within the specified time frame. The Chair of the LSC may recommend withdrawal of volunteers.

Any volunteer who fails to attend for two or more follow-up visits during the study will be deemed to have withdrawn from the study.

If a volunteer withdraws from the study, blood samples collected before their withdrawal from the trial will be used/ stored unless the volunteer specifically requests otherwise.

In all cases of participant withdrawal, excepting those of complete consent withdrawal, long-term safety data collection, including some procedures such as safety bloods, will continue as appropriate if participants have received one or more vaccine doses.

6.4 Compliance with dosing regime

All doses in this vaccine study will be administered by the Investigator and recorded in the CRF. The study medication will be at no time be in the possession of the volunteer and compliance will, therefore, not be an issue.

©Oxford University

6.5 Pregnancy

Should a volunteer become pregnant during the trial, she will be followed up as other volunteers and in addition will be followed until pregnancy outcome. We will not routinely perform venepuncture in a pregnant volunteer.

6.6 Temporary recruitment pause due to COVID-19 pandemic

Recruitment to the trial will be temporarily paused until satisfactory resolution of the COVID-19 public health situation in the UK. Follow up of volunteers will continue to proceed during this time (see 7.4.5).

©Oxford University

7 CLINICAL PROCEDURES

This section describes the clinical procedures for evaluating study participants and follow-up after administration of study vaccine.

7.1 Study procedures

All volunteers will have the same schedule of clinic attendances and procedures as indicated in the schedules of attendance (Table 4) and outlined in Figure 2, including the option of attending for IRV vaccination and follow-up 12 months after enrolment. All participants will receive the ChAdOx2 RabG vaccine and undergo follow-up for a total of 8 weeks. Those who choose to attend the optional IRV vaccination will also receive three doses of IRV and a further 3 weeks of follow-up. The volume of blood donated during the study is outlined in Table 4. Additional visits or procedures may be performed at the discretion of the investigators, e.g., further medical history and physical examination, urine microscopy in the event of positive urinalysis or additional blood tests if clinically relevant.

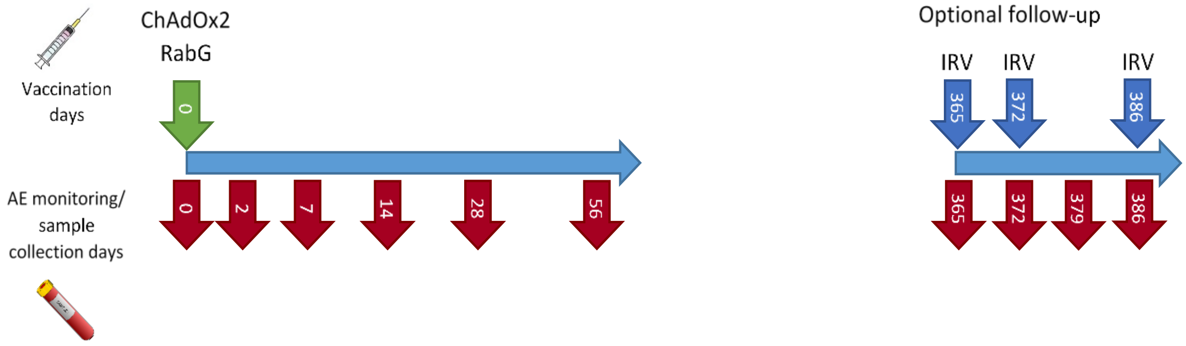

Figure 2. Study schedule of vaccinations and AE monitoring/sample collection. Flexibility in exact timing of certain visits is described in sections 7.4.5 and 7.4.7.

7.2 Observations

Pulse, blood pressure and temperature will be measured at the time-points indicated in the schedule of procedures (Table 4) and may also be measured as part of a physical examination if indicated at other time-points.

7.3 Blood, urine and saliva sampling and analysis

Blood will be drawn for the following laboratory tests and processed:

1. At Oxford University Hospitals NHS Foundation Trust using NHS standard procedures:

- **Haematology;** Full Blood Count

©Oxford University

- **Biochemistry;** Sodium, Potassium, Urea, Creatinine, Albumin, Liver Function Tests (ALT, ALP, Bilirubin)
- **Diagnostic serology;** HBsAg, HCV antibodies, HIV antibodies
- **Immunology;** Human Leukocyte Antigen (HLA) typing

Additional safety blood tests may be performed if clinically relevant at the discretion of the medically qualified investigators. These generally include, but are not limited to AST, GGT and a coagulation screen.

2. At University of Oxford research laboratories:

- **Exploratory Immunology;** Immunogenicity will be assessed by a variety of immunological assays. This may include ex vivo ELISpot assays for interferon gamma and flow cytometry assays, functional antibody assays and B cell analyses. Other exploratory immunological assays including cytokine analysis and other antibody assays, production of monoclonal antibodies, DNA analysis of genetic polymorphisms potentially relevant to vaccine immunogenicity and gene expression studies amongst others may be performed at the discretion of the Investigators.

3. At Wistar Institute, Philadelphia, USA reference laboratories:

- **Rabies virus neutralising assay**.

Urine will be collected for the following tests:

1. At the bedside:

- **Urinalysis;** Urine will be tested for protein, blood and glucose at screening.

2. At Oxford University Hospitals NHS Foundation Trust using NHS standard procedures:

- **β-HCG;** For female volunteers only, urine will be tested for beta-human chorionic gonadotrophin (β-HCG) at screening and immediately prior to each vaccination.

For volunteers consenting to the optional saliva sampling:

Saliva will be collected (by means of a 5mL mouthwash with drinking water) for the following tests:

1. At University of Oxford research laboratories:

- EBV and CMV detection and quantification by quantitative PCR.

©Oxford University

Collaboration with other specialist laboratories in the UK, Europe and outside of Europe for further exploratory immunological tests may occur. This would involve the transfer of serum, plasma, PBMC and/or saliva to these laboratories, but these would remain de-identified. Informed consent for this will be gained from volunteers. Immunological assays will be conducted according to local SOPs.

Participants will be informed that there may be leftover samples of their blood (after all testing for this study is completed), and that such samples may be stored indefinitely for possible future research (exploratory immunology), including genetic analyses to search for correlates of vaccine immunogenicity and efficacy. Participants will be able to decide if they will permit such future use of any leftover samples. With the volunteers' informed consent, any leftover cells, urine and serum/plasma will be frozen indefinitely for future analysis of vaccine-related responses. If a participant elects not to permit this, all of that participant's leftover samples will be discarded in accordance with the HTA and after the required period of storage to meet Good Clinical Practice (GCP) and regulatory requirements.

7.4 Study visits

The study visits and procedures will be undertaken by one of the clinical trials team. The procedures to be included in each visit are documented in the schedule of attendances (Table 4). Each visit is assigned a time-point and a window period, within which the visit will be conducted.

7.4.1 Screening visit

All potential volunteers will have a screening visit, which may take place up to 90 days prior to vaccination. Informed consent will be taken before screening, as described in section 6.2. If consent is obtained, the screening procedures indicated in the schedule of attendances (Table 4) will be undertaken. To avoid unnecessary additional venepuncture, if the appropriate blood test results for screening are available for the same volunteer from a screening visit for another Jenner Institute Clinical Trials group vaccine study, these results may be used for assessing eligibility (provided the results date is within the 3 months preceding enrolment in RAB001).

The participant's general practitioner will be contacted with the written permission of the participant after satisfactory screening as notification that the participant has volunteered for the study and to ascertain any significant medical history. During the screening the volunteers will be asked to provide their National Insurance or passport number so that this can be entered on to a national database which helps prevent volunteers from participating in more than one clinical trial simultaneously or over-volunteering for clinical trials (www.tops.org.uk).

Abnormal clinical findings from the urinalysis or blood tests at screening will be assessed by the lead clinician according to the relevant SOP. Abnormal blood tests following screening will

©Oxford University

be assessed according to site-specific laboratory adverse event grading tables which are filed in the Trial Master File (TMF) or the Investigator Site File (ISF). Any abnormal test result deemed clinically significant may be repeated to ensure it is not a single occurrence. If an abnormal finding is deemed to be clinically significant, the volunteer will be informed and appropriate medical care arranged with the permission of the volunteer.

The eligibility of the volunteer will be reviewed at the end of the screening visit and again when all results from the screening visit have been considered. Decisions to exclude the volunteer from enrolling in the trial or to withdraw a volunteer from the trial will be at the discretion of the Investigator. If eligible, a day 0 visit will be scheduled for the volunteer to receive the vaccine.

7.4.2 Day 0: Enrolment and ChAdOx2 RabG vaccination visit

Volunteers will not be considered enrolled in the study until they have received a vaccine. Before vaccination, the eligibility of the volunteer will be reviewed. Pulse, blood pressure and temperature will be observed and if necessary, a medical history and physical examination may be undertaken to determine need to postpone vaccination depending on criteria listed in section 6.3.6. Vaccinations will be administered as described below in section 7.4.3.

7.4.3 ChAdOx2 RabG vaccinations

Before each vaccination, the on-going eligibility of the volunteer will be reviewed. ChAdOx2 RabG will be administered intramuscularly according to SOP VC002 Vaccination as described in section 8.4. The injection site will be covered with a sterile dressing and the volunteer will stay in the CCVTM for observation, in case of immediate adverse events. Observations will be taken 30 minutes after vaccination (±5 minutes) and the sterile dressing removed and injection site inspected. Observations will also be taken at 60 minutes (±10 minutes), before the volunteer leaves. An oral thermometer, tape measure and diary card (paper or electronic) will be given to each volunteer, with instructions on use, along with a contact card including the emergency 24 hour telephone number to contact the on-call study physician if needed.

Diary cards will collect information on the timing and severity of the following solicited AEs:

Table 3. Solicited AEs as collected on post vaccination diary cards

Local solicited AEs	Systemic solicited AEs
Pain	Fever
Redness	Feverishness
Warmth	Joint pains
Itch	Muscle pains
	Fatigue

©Oxford University

	Headache
	Nausea
	Malaise

Volunteers will be instructed on how to self-assess the severity of these AEs. There will also be space on the diary card to self-document unsolicited AEs, and whether medication was taken to relieve the symptoms.

7.4.4 Sequence of enrolment and ChAdOx2 RabG vaccination of volunteers

The first volunteer in the study will receive 5×10^9 vp of ChAdOx2 RabG (Group 1). They will be vaccinated ahead of any other volunteers and the profile of adverse events will be examined at least 48 hours post-vaccination. Provided there are no safety concerns, as assessed by the CI and Chair of the LSC, a further two volunteers will be vaccinated with the same dose at least 1 hour apart (bringing the total number of volunteers receiving that dose to 3). The CI and the Chair of the LSC will be asked to provide the decision on whether to proceed with vaccination of the first volunteer at the next highest dose (Group 2) following safety review of all Group 1 volunteers. This review will include the results of safety blood tests at day 7 post vaccination.

The first volunteer in the intermediate dose group (Group 2) will receive 2.5×10^{10} vp of ChAdOx2 RabG. They will be vaccinated ahead of any other volunteers and the profile of adverse events will be examined at least 48 hours post-vaccination. Provided there are no safety concerns, as assessed by the CI and Chair of the LSC, a further two volunteers will be vaccinated with the same dose at least 1 hour apart (bringing the total number of volunteers receiving the intermediate dose to 3). The CI and the Chair of the LSC will be asked to provide the decision on whether to proceed with vaccination of the first volunteer at the next highest dose (Group 3) following safety review of all Group 2 volunteers. This review will include the results of safety blood tests at day 7 post vaccination.

The first volunteer in the highest dose group (Group 3) will receive 5×10^{10} vp of ChAdOx2 RabG. This volunteer will be vaccinated ahead of any other volunteers and the profile of adverse events will be examined at least 48 hours post-vaccination. Provided there are no safety concerns, as assessed by the CI and Chair of the LSC, a further two volunteers will be vaccinated with the same dose at least 1 hour apart (bringing the total number of volunteers receiving the highest dose to 3). The CI and the Chair of the LSC will be asked to provide the decision on whether to proceed with vaccination of the remaining volunteers at this dose following safety review of the first three Group 3 volunteers. This review will include the results of safety blood tests at day 7 post vaccination.

Should the CI and Chair of the LSC have safety concerns during any safety review, holding rules may be activated (see section 9.9) or they may decide to vaccinate more volunteers in Groups 1 or 2, up to a maximum of 6 volunteers per group. This would be to ensure

©Oxford University

confidence in safety prior to vaccinating volunteers at higher doses, and that 6 volunteers receive the preferred dose identified during this trial.

7.4.5 Subsequent visits during core study period: days 2, 7, 14, 28, 56

Follow-up visits will take place 2 (±1), 7 (±2), 14 (±3), 28 (±3), and 56 (±7) days after vaccination with ChAdOx2 RabG. Volunteers will be assessed for local and systemic adverse events, interim history, physical examination, review of diary cards (paper or electronic) and blood tests at these time points as detailed in the schedule of attendances Table 4. Blood will also be taken for exploratory immunology purposes as indicated in Table 4.

If volunteers experience adverse events (laboratory or clinical), which the investigator (physician), CI and/or Chair of the LSC determine necessary for further close observation, the volunteer may be admitted to an NHS hospital for observation and further medical management under the care of the Consultant on call.

Where possible, and with their permission, volunteer follow up visits may take place in their own homes during the COVID-19 public health crisis. These will be carried out in accordance with relevant Jenner SOPs. In exceptional circumstances, other appropriately risk assessed Jenner institute facilities may be used to see asymptomatic volunteers for follow up visits including: phlebotomy rooms at the Old Road Campus Research Build, Roosevelt Dr, Headington, Oxford.

Depending on the public health measures in place, Day 56 follow up visits may take place via phone as primary safety endpoint data for this visit may be adequately captured in this way.

7.4.6 Optional IRV vaccination and follow-up

For volunteers that choose to return for the optional IRV vaccination, vaccination following the Green Book recommended schedule for PrEP (7) and simulating PEP in this study, will take place on days 365, 372 and 386. A follow-up visit will also take place on day 379. The visits on days 372, 379, and 386 are linked to the visit on day 365, and will take place 7 (±0), 14 (±3) and 21 (+7) days following the actual date of the day 365 visit. Volunteers will be assessed for local and systemic adverse events, interim history, and physical examination, at these time points as detailed in the schedule of attendances Table 4. Blood will also be taken for exploratory immunology purposes as indicated in Table 4 and outlined in section 3.14.

If volunteers experience adverse events (laboratory or clinical), which the investigator (physician), CI and/or Chair of the LSC determine necessary for further close observation, the volunteer may be admitted to an NHS hospital for observation and further medical management under the care of the Consultant on call.

©Oxford University

7.4.7 Schedule of attendances

Table 4. Schedule of attendances

	Core visits							Optional visits			
Attendance Number (visit)	1[S]	2	3	4	5	6	7	8	9	10	11
Timeline (days)	≤ 90	0	2	7	14	28	56	365	372*	379*	386*
Time window (days)			±1	±2	±3	±3	±7	±60	±0*	±3*	+7*
Informed Consent	X										
Review contraindications, inclusion and exclusion criteria	X	X						X			
ChAdOx2 RabG vaccination		X									
IRV vaccination								X	X		X
Vital signs	X	X	X	X	X	X	X	X	X	X	X
Ascertainment of adverse events		X	X	X	X	X	X	X	X	X	X
Diary cards provided		X									
Diary cards collected						X					
Medical History, Physical Examination	X	(X)	(X)	(X)	(X)	(X)	(X)	(X)	(X)	(X)	(X)
Photography of injection site		(X)	(X)	(X)	(X)	(X)	(X)	(X)	(X)	(X)	(X)
Urinalysis	X										
Urinary β–HCG (women only)	X	X						X			
(Optional) saliva collection	X	X	X	X	X	X	X	X	X	X	X
HBsAg, HCV Ab, HIV serology (mL)	5										
HLA typing (mL)		4									
Biochemistry, Haematology (mL)	5	5	5	5		5					
Exploratory immunology (mL)		50		50	50	50	50	50	50	50	50
Blood volume per visit	10	59	5	55	50	55	50	50	50	50	50
Cumulative blood volume	10	69	74	129	179	234	284	334	384	434	484

[S] screening visit.
X indicates carried out at visit.
(X) indicates carried out at visit if considered necessary.
*The timelines for visits 9, 10 and 11 are related to visit 8, and will take place 7±0, 14±3 and 21+7 days following visit 8 respectively.
Shaded cells indicate optional parts of the study.

©Oxford University

8 INVESTIGATIONAL PRODUCTS

All participants will receive one vaccination with ChAdOx2 RabG as outlined in Figure 2 and Table 4. The dose received will depend on the Group, as outlined in Section 5.2.

Participants may also receive three doses of IRV (Rabies Vaccine BP, Rabipur, or Verorab) following the Green Book recommendations (7) as outlined in Figure 2 and Table 4.

8.1 Manufacturing and Presentation

8.1.1 ChAdOx2 RabG

ChAdOx2 RabG has been developed and produced by the Jenner Institute, University of Oxford. The ChAdOx2 RabG vaccine consists of the replication-deficient simian adenovirus vector ChAdOx2, containing glycoprotein of rabies expressed from the strong CMV IE promoter.

ChAdOx2 RabG is manufactured in formulation buffer at a concentration of $>1.1 \times 10^{11}$ vp/mL. The drug product is filled into 2mL glass vials with a 13 mm grey bromobutyl rubber freeze-dry stopper (CE Marked, supplied by Adelphi Tubes) and a 13 mm aluminium seal. The nitrogen filled vials are supplied sterile. The containers and closures are tested for compliance with defined specifications. The vials are made from Ph Eur Type 1 glass.

8.1.2 IRVs

Rabies Vaccine BP is manufactured by Sanofi Pasteur. The vaccine is supplied as freeze-dried powder and solvent for suspension and for injection. The powder is pinkish beige to orangey yellow. The solvent is a clear, colourless solution. Following reconstitution with the solvent supplied, the suspension will be a pinkish colour and free from particles.

Rabipur is manufactured by GSK Vaccines. The vaccine is supplied as freeze-dried powder and solvent for suspension and for injection. The powder is white. The solvent is a clear, colourless solution. Following reconstition with the solvent supplied, the suspension will be a clear-colourless solution and free from particles.

Verorab is manufactured by Sanofi Pasteur. The vaccine is supplied as freeze-dried powder and solvent for suspension and for injection. The powder is white. The solvent is a clear, colourless solution. Following reconstition with the solvent supplied, the suspension will be a clear-colourless solution and free from particles.

8.2 Supply

8.2.1 ChAdOx2 RabG

ChAdOx2 RabG has been manufactured under Good Manufacturing Practice (GMP) conditions at the Clinical Biomanufacturing Facility (CBF), University of Oxford. At the CBF the vaccine will be certified and labelled for the trial by a Qualified Person (QP) before transfer to the clinical site.

©Oxford University

8.2.2 IRVs

There will be no trial-specific labelling or QP release for IRVs, which will be assembled on site according to the applicable SmPC.

Rabies Vaccine BP and Verorab is manufactured by Sanofi Pasteur.

Rabipur is manufactured is manufactured by GSK Vaccines.

All IRVs will be supplied by Oxford University Hospitals Pharmacy.

8.3 Storage

8.3.1 ChAdOx2 RabG

ChAdOx2 RabG is stored at nominal -80°C in a locked freezer, at the clinical site. All movements of the study vaccines will be documented in accordance with existing standard operating procedure (SOP). Vaccine accountability, storage, shipment and handling will be in accordance with relevant SOPs and forms.

8.3.2 IRVs

IRVs will be stored between +2°C and +8°C until just prior to administration, in accordance with the applicable SmPC. The will be stored in a locked fridge at the clinical site. All movements of the study vaccines will be documented in accordance with existing standard operating procedure (SOP). Vaccine accountability, storage, shipment and handling will be in accordance with relevant SOPs and forms.

8.4 Administration of Investigational Medicinal Products

On vaccination day, ChAdOx2 RabG will be allowed to thaw to room temperature and will be administered within 1 hour of removal from the freezer. The vaccine will be administered intramuscularly into the deltoid of the non-dominant arm (preferably). All volunteers will be observed in the unit for 1 hour (±10 minutes) after vaccination. During administration of the investigational products, Advanced Life Support drugs and resuscitation equipment will be immediately available for the management of anaphylaxis. Vaccination will be performed and the IMPs handled according to the relevant SOPs.

IRVs will be administered following the applicable SmPC.

8.5 Minimising environmental contamination with genetically modified organisms (GMO)

The study will be performed in accordance with UK Genetically Modified Organisms (Contained Use) Regulations (2014). In order to minimise dissemination of the recombinant vectored vaccine virus into the environment, ChAdOx2 RabG inoculation sites will be covered with a dressing after immunisation. This should absorb any virus that may leak out through

©Oxford University

the needle track. The dressing will be removed from the injection site after 30 minutes (+15/-5 minutes) and will be disposed as GMO waste by autoclaving.

©Oxford University

9 ASSESSMENT OF SAFETY

Safety will be assessed by the frequency, incidence and nature of adverse events and serious adverse events arising during the study.

9.1 Definitions

9.1.1 Adverse Event (AE)

An AE is any untoward medical occurrence in a volunteer, which may occur during or after administration of an IMP and does not necessarily have a causal relationship with the intervention. An AE can therefore be any unfavourable and unintended sign (including an abnormal laboratory finding), symptom or disease temporally associated with the study intervention, whether or not considered related to the study intervention.

9.1.2 Adverse Reaction (AR)

An AR is any untoward or unintended response to an IMP. This means that a causal relationship between the IMP and an AE is at least a reasonable possibility, i.e., the relationship cannot be ruled out. All cases judged by the reporting medical Investigator as having a reasonable suspected causal relationship to an IMP (i.e. possibly, probably or definitely related to an IMP) will qualify as adverse reactions.

9.1.3 Serious Adverse Event (SAE)

An SAE is an AE that results in any of the following outcomes, whether or not considered related to the study intervention.

- Death
- Life-threatening event (i.e., the volunteer was, in the view of the Investigator, at immediate risk of death from the event that occurred). This does not include an AE that, if it occurred in a more severe form, might have caused death.
- Persistent or significant disability or incapacity (i.e., substantial disruption of one's ability to carry out normal life functions).
- Hospitalisation, regardless of length of stay, even if it is a precautionary measure for continued observation. Hospitalisation (including inpatient or outpatient hospitalisation for an elective procedure) for a pre-existing condition that has not worsened unexpectedly does not constitute a serious AE.
- An important medical event (that may not cause death, be life threatening, or require hospitalisation) that may, based upon appropriate medical judgment, jeopardise the volunteer and/or require medical or surgical intervention to prevent one of the outcomes listed above. Examples of such medical events include allergic reaction requiring intensive treatment in an emergency room or clinic, blood dyscrasias, or convulsions that do not result in inpatient hospitalisation.
- Congenital anomaly or birth defect.

©Oxford University

9.1.4 Serious Adverse Reaction (SAR)

An adverse event (expected or unexpected) that is both serious and, in the opinion of the reporting Investigator or Sponsors, believed to be possibly, probably or definitely due to an IMP or any other study treatments, based on the information provided.

9.1.5 Suspected Unexpected Serious Adverse Reaction (SUSAR)

A serious adverse reaction, the nature and severity of which is not consistent with the information about the medicinal product in question set out in the Investigator's Brochure (IB) or Summary of Product Characteristics (SmPC).

9.2 Causality assessment

For every AE, an assessment of the relationship of the event to the administration of the vaccine will be undertaken by the CI-delegated clinician. An intervention-related AE refers to an AE for which there is a probable or definite relationship to administration of a vaccine. An interpretation of the causal relationship of the intervention to the AE in question will be made, based on the type of event; the relationship of the event to the time of vaccine administration; and the known biology of the vaccine therapy (Table 5). Alternative causes of the AE, such as the natural history of pre-existing medical conditions, concomitant therapy, other risk factors and the temporal relationship of the event to vaccination will be considered and investigated. Causality assessment will take place during planned safety reviews, interim analyses (e.g. if a holding rule is activated) and at the final safety analysis, except for SAEs, which should be assigned by the reporting investigator.

Table 5. Guidelines for assessing the relationship of vaccine administration to an AE.

0	**No Relationship**	No temporal relationship to study product; ***and***<br>Alternate aetiology (clinical state, environmental or other interventions); ***and***<br>Does not follow known pattern of response to study product.
1	**Unlikely**	Unlikely temporal relationship to study product; ***and***<br>Alternate aetiology likely (clinical state, environmental or other interventions); ***and***<br>Does not follow known typical or plausible pattern of response to study product.
2	**Possible**	Reasonable temporal relationship to study product; ***or***<br>Event not readily produced by clinical state, environmental or other interventions; ***or***<br>Similar pattern of response to that seen with other vaccines.

©Oxford University

3	**Probable**	Reasonable temporal relationship to study product; ***and***<br>Event not readily produced by clinical state, environment, or other interventions; ***or***<br>Known pattern of response seen with other vaccines.
4	**Definite**	Reasonable temporal relationship to study product; ***and***<br>Event not readily produced by clinical state, environment, or other interventions; ***and***<br>Known pattern of response seen with other vaccines.

9.3 Expectedness assessment

Expectedness for ChAdOx2 RabG will be determined according to the information set out in the IB. As no SARs are expected for ChAdOx2 RabG, any SARs associated with its administration will be reported as SUSARs.

Expectedness for IRVs will be determined according to the information set out in the applicable SmPC. Because volunteers receiving IRV will also have received ChAdOx2 RabG, around one year earlier, any SARs occurring after IRV administration will also be reported as SUSARs relating to ChAdOx2 RabG if, in the opinion of the Chair of the LSC, they are at least possibly related to ChAdOx2 RabG.

9.4 Reporting procedures for all Adverse Events (see SOP VC027)

All local and systemic AEs occurring in the 28 days following each IMP vaccination observed by the Investigator or reported by the volunteer, whether or not attributed to study medication, will be recorded (excluding those expected consequences from venepuncture, described in section 5.6). Recording and reporting of all AEs will take place as detailed in SOP VC027. All AEs that result in a volunteer's withdrawal from the study will be followed up until a satisfactory resolution occurs, or until a non-study related causality is assigned (if the volunteer consents to this). Serious adverse events (SAEs) will be collected throughout the entire trial period.

9.4.1 Reporting procedures for SAEs (see SOP OVC005 Safety Reporting)

In order to comply with current regulations on serious adverse event reporting to regulatory authorities, the event will be documented accurately and notification deadlines respected. SAEs will be reported on the SAE forms to members of the study team immediately once an Investigator becomes aware of their occurrence, as described in SOP OVC005. Copies of all reports will be forwarded for review to the CI (as the Sponsor's representative) within 24 hours of the Investigator being aware of the suspected SAE. The Chair of the LSC will be notified of SAEs that are deemed possibly, probably or definitely related to study interventions within 24 hours of the Investigator being aware of their occurrence. SAEs will

©Oxford University

not normally be reported immediately to the ethics committee(s) unless there is a clinically important increase in occurrence rate, an unexpected outcome, or a new event that is likely to affect safety of trial volunteers, at the discretion of the CI and/or Chair of the LSC. In addition to the expedited reporting above, the Investigator shall include all SAEs in the annual Development Safety Update Report (DSUR).

9.4.2 Reporting Procedures for SUSARS

The CI will report all SUSARs to the Medicines and Healthcare products Regulatory Authority (MHRA) and ethical committee(s) within required timelines (15 calendar days for all SUSARs from the date of awareness, unless life threatening in which case 7 days, with a final report within a further 8 days (total 15)). The CI will also inform all Investigators concerned of relevant information about SUSARs that could adversely affect the safety of participants. All SUSARs and deaths occurring during the study will be reported to the Sponsor. For all deaths, available autopsy reports and relevant medical reports will be made available for reporting to the relevant authorities.

9.4.3 Development Safety Update Report

The CI will submit (in addition to the expedited reporting above) Development Safety Update Reports (DSURs) once a year throughout the clinical trial, or on request, to the Competent Authority (MHRA in the UK), Ethics Committee, HRA (where required), Host NHS Trust and Sponsor.

9.5 Assessment of severity

The severity of clinical and laboratory adverse events will be assessed according to the scales in Table 6, Table 7 and Table 8, also described in the SOP VC027.

Table 6. Severity grading criteria for local adverse events for IM injections

Adverse Event	Grade	Intensity
Erythema at injection site*	1	>3 - ≤50 mm
	2	>50 - ≤100 mm
	3	>100 mm
Swelling at injection site	1	>3 - ≤20 mm
	2	>20 - ≤50 mm
	3	>50 mm

©Oxford University

Ulceration/necrosis of skin at injection site	1	-
	2	-
	3	Any

*erythema or swelling ≤3mm is an expected consequence of skin puncture and will therefore not be considered an adverse event.

Table 7. Severity grading criteria for physical observations

	Grade 1 (mild)	Grade 2 (moderate)	Grade 3 (severe)
Fever (oral)	37.6°C - 38.0°C	38.1°C – 39.0°C	>39.0°C
Tachycardia (bpm)*	101 - 115	116 – 130	>130
Bradycardia (bpm)**	50 – 54	40 – 49	<40
Systolic hypertension (mmHg)	141 - 159	160 – 179	≥180
Systolic hypotension (mmHg)***	85 - 89	80 – 84	<80
Diastolic hypertension (mmHg)	91 - 99	100 – 109	≥110

* Taken after ≥10 minutes at rest

** Use clinical judgement when characterising bradycardia among some healthy participant populations, for example, conditioned athletes.

*** Only if symptomatic (e.g. dizzy/ light-headed)

Table 8. Severity grading criteria for local and systemic AEs.

**GRADE 0**	None: Symptom not experienced
**GRADE 1**	Mild: Short-lived or mild symptoms; medication may be required. No limitation to usual activity
**GRADE 2**	Moderate: Mild to moderate limitation in usual activity. Medication may be required.
**GRADE 3**	Severe: Considerable limitation in activity. Medication or medical attention required.

9.6 Procedures to be followed in the event of abnormal findings

Laboratory parameters for inclusion/exclusion in the trial will be considered on an individual basis, with investigator discretion for interpretation of results and the need for repeated tests. In general, volunteers will be excluded if a result at screening constitutes what would qualify as a grade 1 (or higher) laboratory adverse event, according to the site-specific

©Oxford University

laboratory adverse event tables (stored in the TMF and/or ISF, also available in SOP VC027). Abnormal clinical findings from medical history, examination or blood tests will be assessed as to their clinical significance throughout the trial. If a test is deemed clinically significant, it may be repeated, to ensure it is not a single occurrence. If a test remains clinically significant, the volunteer will be informed and appropriate medical care arranged as appropriate and with the permission of the volunteer. Decisions to exclude the volunteer from enrolling in the trial or to withdraw a volunteer from the trial will be at the discretion of the Investigator.

9.7 Local Safety Committee

A Local Safety Committee (LSC) will be appointed to provide real-time safety oversight. The LSC will review SAEs deemed possibly, probably or definitely related to study interventions. The LSC will be notified within 24 hours of the Investigators' being aware of their occurrence. The LSC has the power to place the study on hold if deemed necessary following a study intervention-related SAE. At the time of writing the LSC will be chaired by Dr Brian Angus, a Clinical Tutor in Medicine, Honorary Consultant Physician and Director, Oxford Centre for Clinical Tropical Medicine at the University of Oxford.

The Chair of the LSC may be contacted for advice and independent review by the Investigator or trial Sponsor in the following situations:

- Following any SAE deemed to be possibly, probably, or definitely related to a study intervention.
- Any other situation where the Investigator or trial Sponsor feels independent advice or review is important.

9.8 Interim safety reviews

The safety profile of the IMP will be assessed on an on-going basis by the Investigators with communication to the LSC as necessary. The CI and relevant Investigators (as per the trial delegation log) will also review safety issues and SAEs as they arise.

The following interim safety reviews will take place;

- 48 hours following the IMP vaccination of the first volunteer in each group, the CI and Chair of the LSC will decide on whether to proceed with vaccination of the next 2 volunteers in the group;
- Following vaccination of the first 3 volunteers in each group, the CI and Chair of the LSC will decide on whether to proceed to the next highest dose group (if applicable) or vaccinate another 3 volunteers at the same dose, or vaccinate another 3 volunteers at the next lowest dose. This review will include the results of safety blood tests at day 7 post vaccination.

©Oxford University

9.9 Safety Holding Rules

Safety holding rules have been developed considering the fact that this is a first-in-human dose escalation study.

'Solicited adverse events' are those listed as potentially foreseeable risks in section 5.6 of the protocol, occurring within the first 7 days after vaccination (day of vaccination and six subsequent days). 'Unsolicited adverse events' are adverse events other than the foreseeable AEs occurring within the first 7 days, or any AEs occurring after the first 7 days after vaccination.

9.9.1 Group holding rules

For safety reasons the first volunteer to receive a new vaccine dose will be vaccinated alone and we will wait 48 hours before vaccinating subsequent volunteers. Two further volunteers may be vaccinated 48 hours after the first, and then at least another 7 day gap will be left before vaccinating any further volunteers receiving the same dose of the vaccine.

The group holding rules are as follows;

- **Solicited local adverse events:**
    - If 2 or more vaccinations in a group are followed by the same Grade 3 solicited local adverse event beginning within 2 days after vaccination (day of vaccination and one subsequent day) and persisting at Grade 3 for >48 hrs.

- **Solicited systemic adverse events:**
    - If 2 or more vaccinations in a group are followed by the same Grade 3 solicited systemic adverse event beginning within 2 days after vaccination (day of vaccination and one subsequent day) and persisting at Grade 3 for >48 hrs.

- **Unsolicited adverse events:**
    - If 2 or more vaccinations in a group are followed by the same Grade 3 unsolicited adverse event (including the same laboratory adverse event) that is considered possibly, probably or definitely related to vaccination and persists at Grade 3 for > 48hrs.

- **A serious adverse event considered possibly, probably or definitely related to vaccination occurs**

- **Death occurs**

- **A life-threatening reaction occurs**

©Oxford University

If a holding rule has been met we will inform the regulatory authority; following an internal safety review, if it is deemed appropriate to restart dosing, a request to restart dosing with pertinent data must be submitted to the regulatory authority as a request for a substantial amendment. The internal safety review will consider:

- The relationship of the AE or SAE to the vaccine.
- The relationship of the AE or SAE to the vaccine dose, or other possible causes of the event.
- If appropriate, additional screening or laboratory testing for other volunteers to identify those who may develop similar symptoms and alterations to the current Participant Information Sheet (PIS).
- New, relevant safety information from ongoing research programs on the various components of the vaccine.

The sponsor, LSC, and vaccine manufacturers will also be notified if a holding rule is activated or released.

As per section 6.3.6, if a volunteer has an acute illness (moderate or severe illness with or without fever) or a fever (oral temperature greater than 37.5°C) at the scheduled time of administration of investigational product, the volunteer will not receive the vaccine at that time. The vaccine may be administered to that volunteer at a later date within the time window specified in the protocol (see Table 4) or they may be withdrawn from the study at the discretion of the Investigator.

All vaccinated volunteers will be followed for safety until the end of their planned participation in the study or until resolution or stabilisation (if determined to be chronic sequelae) of their AEs, providing they consent to this.

In addition to these pre-defined criteria, the study can be put on hold upon advice of the LSC, CI, Study Sponsor, Regulatory Authority or Ethical Committee(s), for any single event or combination of multiple events which, in their professional opinion, jeopardise the safety of the volunteers or the reliability of the data.

©Oxford University

10 DATA MANAGEMENT

10.1 Data Handling

The Chief Investigator will be responsible for all data that accrues from the study. The data will be entered into the volunteers' CRFs in a paper and/or electronic format (using OpenClinica™ database). Electronic data will be stored on secure servers which are outsourced by OpenClinica™. Data will be entered into the OpenClinica Database via a secure web browser. OpenClinica™ meets FDA part 11B standards. This includes safety data, laboratory data (both clinical and immunological) and outcome data.

Adverse event data will also be entered onto electronic or paper diaries by the volunteer

10.2 Record Keeping

The Investigators will maintain appropriate medical and research records for this trial, in compliance with GCP and regulatory and institutional requirements for the protection of confidentiality of volunteers. The Chief Investigator, co-Investigators and clinical research nurses will have access to records. The Investigators will permit authorised representatives of the Sponsor(s), as well as ethical and regulatory agencies to examine (and when required by applicable law, to copy) clinical records for the purposes of quality assurance reviews, audits and evaluation of the study safety and progress.

10.3 Source Data and Case Report Forms (CRFs)

All protocol-required information will be collected in CRFs designed by the Investigator. All source documents will be filed in the CRF. Source documents are original documents, data, and records from which the volunteer's CRF data are obtained. For this study, these will include, but are not limited to, volunteer consent form, blood results, GP response letters, laboratory records, ediaries, and correspondence. In the majority of cases, CRF entries will be considered source data as the CRF is the site of the original recording (i.e. there is no other written or electronic record of data). In this study this will include, but is not limited to medical history, medication records, vital signs, physical examination records, urine assessments, blood results, adverse event data and details of vaccinations. All source data and volunteer CRFs will be stored securely.

10.4 Data Protection

The study protocol, documentation, data and all other information generated will be held in strict confidence. No information concerning the study or the data will be released to any unauthorised third party, without prior written approval of the sponsor.

©Oxford University

10.5 Data Quality

Data collection tools will undergo appropriate validation to ensure that data is collected accurately and completely. Datasets provided for analysis will be subject to quality control processes to ensure analysed data is a true reflection of the source data.

Trial data will be managed in compliance with local data management SOPs (including the overarching SOP OVC007 Data and Database Management). If additional, study specific information is required, an approved Data Management Plan will be implemented.

The trial will comply with the General Data Protection Regulation (GDPR) and Data Protection Act 2018, which requires data to be de-identified as soon as it is practical to do so.

©Oxford University

11 STATISTICS

Statistical analysis will be appropriate to the study's primary aim, i.e. to provide a descriptive and preliminary assessment of the safety of ChAdOx2 RabG, with limited, highly preliminary immunological information being sought as a subsidiary aim.

Appropriate descriptive statistics and graphical representations will be used to present the safety and immunogenicity data, similar to those used in similar previous studies (35).

No comparisons will be made and as such no statistical inference testing will be performed.

11.1 Sample Size Selection

The sample size is chosen in line with the primary objective of providing adequate descriptive safety information to permit further clinical evaluation. The next step of the clinical development plan is a further Phase I study, initially in Tanzanian adults. The key determinant of the sample size is thus expert opinion regarding the extent of data needed for this study. Our Tanzanian collaborator, in discussion with the Tanzanian FDA, has advised that a total of 12 vaccinees, with at least 6 at the preferred dose level, would be regarded as adequate.

The secondary objective (measurement of induction of virus neutralising antibody) will also involve descriptive analysis only, without any comparisons being made. We will not seek to formally quantify the dose - immunogenicity relationship within this trial. Given that no statistical inference testing will be performed, no statistical sample size / power calculation is appropriate.

©Oxford University

12 ETHICS AND REGULATORY CONSIDERATIONS

12.1 Declaration of Helsinki

The Investigators will ensure that this study is conducted according to the principles of the Declaration of Helsinki (2008).

12.2 Guidelines for Good Clinical Practice

The Investigators will ensure that this study is conducted in full conformity with the Good Clinical Practice (GCP), the requirements of the Medicines for Human Use (Clinical Trials) Regulations 2004, and local regulatory requirements.

12.3 Approvals

The protocol, informed consent form, participant information sheet and any proposed advertising material will be submitted to an appropriate Research Ethics Committee (REC), HRA (where required), regulatory authorities (MHRA in the UK), and host institution(s) for written approval.

The Investigator will submit and, where necessary, obtain approval from the above parties for all substantial amendments to the original approved documents.

No substantial amendments to this protocol will be made without consultation with, and agreement of, the Sponsor. Any substantial amendments to the trial that appear necessary during the course of the trial must be discussed by the Investigator and Sponsor concurrently. If agreement is reached concerning the need for an amendment, it will be produced in writing by the Chief Investigator and will be made a formal part of the protocol following ethical and regulatory approval.

The Investigator is responsible for ensuring that changes to an approved trial, during the period for which regulatory and ethical committee(s) approval has already been given, are not initiated without regulatory and ethical committee(s)' review and approval except to eliminate apparent immediate hazards to the participant.

12.4 Reporting

The CI shall submit once a year throughout the clinical trial, or on request, an Annual Progress Report to the REC, HRA (where required), host organisation, funder (where required) and Sponsor. In addition, an End of Trial notification and final report will be submitted to the MHRA, the REC, host organisation and Sponsor.

©Oxford University

12.5 Volunteer Confidentiality

All data will be de-identified: volunteer data will be identified by a unique study number in the CRF and database. A separate confidential file containing identifiable information will be stored in a secured location in accordance with the Data Protection Act 2018. All documents will be stored securely and only accessible by study staff and authorised personnel. The study staff will safeguard the privacy of participants' personal data. Photographs taken of vaccination sites (if required, with the volunteer's written, informed consent) will not include the volunteer's face and will be identified by the date, trial code and participant's unique identifier. Once developed, photographs will be stored as confidential records, as above. This material may be shown to other professional staff, used for educational purposes, or included in a scientific publication.

We will keep identifiable information about volunteers such as contact details for a minimum of 5 years after the study has finished. The need to store this information for longer in relation to licensing of the vaccine will be subject to ongoing review. For effective vaccines that may be licensed, we may store research data securely for at least 15 years after the end of the study, subject to adjustments in clinical trials regulations. In addition to the de-identified scientific data, we will also store documents containing personal information volunteers provide when registering for the trial (including contact details), medical information and signed consent forms during this archiving period.

The study team will use volunteer name and contact details, to contact them about the research study, and make sure that relevant information about the study is recorded for their care, in relation to their health during the study and to oversee the quality of the study. At the completion of the study, unless volunteers consent otherwise (e.g. if they request to be informed of other trials), volunteer personal details will not be used to contact them other than in exceptional circumstances concerning their safety. If they consent to take part in another study carried out by the Jenner Institute, personal information and medical information including blood test results may be accessed to avoid unnecessary repetition.

A photocopy of volunteer ID (driving licence, passport or national ID card) will be taken at the screening visit and retained until the end of the study.

Volunteer bank details will be stored for 7 years in line with university financial policy.

©Oxford University

13 QUALITY CONTROL AND QUALITY ASSURANCE PROCEDURES

13.1 Investigator procedures

Approved site-specific standard operating procedures (SOPs) will be used at all clinical and laboratory sites.

13.2 Monitoring

Monitoring will be performed according to ICH GCP by Clinical Trials Research Governance (CTRG). Following written SOPs, the monitors will verify that the clinical trial is conducted and data are generated, documented and reported in compliance with the protocol, GCP and the applicable regulatory requirements. The Investigator site will provide direct access to all trial related source data/documents and reports for the purpose of monitoring and auditing by the Sponsor and inspection by local and regulatory authorities.

13.3 Protocol deviation

Any deviations from the protocol will be documented in a protocol deviation form and filed in the trial master file. Each deviation will be assessed as to its impact on volunteer safety and study conduct. Significant deviations will be listed in the end of study report.

13.4 Audit & inspection

The QA manager conducts systems based internal audits to check that trials are being conducted according to local procedures and in compliance with GCP and applicable regulations.

The Sponsor, trial sites, and ethical committee(s) may carry out audits to ensure compliance with the protocol, GCP and appropriate regulations.

GCP inspections may also be undertaken by the MHRA to ensure compliance with protocol and the Medicines for Human Use (Clinical Trials) Regulations 2004, as amended. The Sponsor will assist in any inspections and will support the response to the MHRA as part of the inspection procedure.

©Oxford University

14 FINANCING AND INSURANCE

14.1 Financing

The study is funded by the UK Medical Research Council.

14.2 Insurance

The University has a specialist insurance policy in place which would operate in the event of any participant suffering harm as a result of their involvement in the research (Newline Underwriting Management Ltd, at Lloyd's of London).

14.3 Compensation

Volunteers will be compensated for their time and for the inconvenience caused by procedures. They will be compensated £25 for attending the screening visit. For all other trial visits as outlined in Table 4, compensation will be calculated according to the following:

- Travel expenses:
  - £15 per visit. Where travel expenses are greater than £10 per visit because the volunteer lives outside the city of the trial site, the volunteer will be given further reimbursement to meet the cost of travel necessary for study visits following receipt of valid proof of costs incurred.

- Inconvenience of blood tests:
  - £10 per blood donation

- Time required for visit:
  - £20 per hour

The total amount compensated will be approximately £335 per enrolled volunteer, depending on the exact number of visits and whether any repeat or additional visits are necessary. Volunteers attending the option IRV vaccination and follow up will receive a further payment of approximately £270, giving total payments of approximately £605.

©Oxford University

15 SERIOUS BREACHES

The Medicines for Human Use (Clinical Trials) Regulations contain a requirement for the notification of "serious breaches" to the MHRA within 7 days of the Sponsor becoming aware of the breach.

A serious breach is defined as “A breach of GCP or the trial protocol which is likely to affect to a significant degree

(a) the safety or physical or mental integrity of the participants of the trial; or

(b) the scientific value of the trial”.

In the event that a serious breach is suspected the Sponsor will be informed within one working day.

©Oxford University

16 PUBLICATION POLICY

The Investigators will be involved in reviewing drafts of the manuscripts, abstracts, press releases and any other publications arising from the study. Data from the study may also be used as part of a thesis for a PhD or MD.

©Oxford University

17 REFERENCES

1. Fooks AR, Cliquet F, Finke S, Freuling C, Hemachudha T, Mani RS, et al. Rabies. Nature Reviews Disease Primers. 2017;3:17091.

2. World Health Organization. WHO Expert Consultation on Rabies. Third report. World Health Organization technical report series. 2018(1012):1-184, back cover.

3. Changalucha J, Steenson R, Grieve E, Cleaveland S, Lembo T, Lushasi K, et al. The need to improve access to rabies post-exposure vaccines: Lessons from Tanzania. Vaccine. 2018.

4. Hampson K, Coudeville L, Lembo T, Sambo M, Kieffer A, Attlan M, et al. Estimating the global burden of endemic canine rabies. PLoS neglected tropical diseases. 2015;9(4):e0003709.

5. O'Brien KL, Nolan T. The WHO position on rabies immunization - 2018 updates. Vaccine. 2018;Epub ahead of print.

6. World Health O. Rabies vaccines: WHO position paper, April 2018 - Recommendations. Vaccine. 2018;36(37):5500-3.

7. Department of Health. Immunisation against infectious disease (Green Book). Chapter 27: Rabies. In: Health Do, editor. London: Department of Health; 2018.

8. Jonker EFF, Visser LG. Single visit rabies pre-exposure priming induces a robust anamnestic antibody response after simulated post-exposure vaccination: results of a dose-finding study. Journal of travel medicine. 2017;24(5).

9. Khawplod P, Jaijaroensup W, Sawangvaree A, Prakongsri S, Wilde H. One clinic visit for pre-exposure rabies vaccination (a preliminary one year study). Vaccine. 2012;30(19):2918-20.

10. Soentjens P, De Koninck K, Tsoumanis A, Herssens N, Van Den Bossche D, Terryn S, et al. A Comparative Immunogenicity and Safety Trial of Two Different Schedules of Single-visit Intradermal Rabies Post-exposure Vaccination Following a Single-visit Pre-exposure Vaccination. Clin Infect Dis. 2018;Epub ahead of print.

11. Lembo T, Hampson K, Kaare MT, Ernest E, Knobel D, Kazwala RR, et al. The feasibility of canine rabies elimination in Africa: dispelling doubts with data. PLoS neglected tropical diseases. 2010;4(2):e626.

12. Control GAfR. Policy and Advocacy: Global Alliance for Rabies Control; 2015 [Available from: https://rabiesalliance.org/policy.

13. World Health Organization. Rabies: rationale for investing in the global elimination of dog-mediated human rabies. 2015.

14. Chulasugandha P, Khawplod P, Havanond P, Wilde H. Cost comparison of rabies pre-exposure vaccination with post-exposure treatment in Thai children. Vaccine. 2006;24(9):1478-82.

15. Wang C, Dulal P, Zhou X, Xiang Z, Goharriz H, Banyard A, et al. A simian-adenovirus-vectored rabies vaccine suitable for thermostabilisation and clinical development for low-cost single-dose pre-exposure prophylaxis. PLoS neglected tropical diseases. 2018;12(10):e0006870.

16. Xiang ZQ, Greenberg L, Ertl HC, Rupprecht CE. Protection of non-human primates against rabies with an adenovirus recombinant vaccine. Virology. 2014;450-451(1096-0341 (Electronic)):243-9.

17. Bett AJ, Haddara W, Prevec L, Graham FL. An efficient and flexible system for construction of adenovirus vectors with insertions or deletions in early regions 1 and 3. Proc Natl Acad Sci U S A. 1994;91(19):8802-6.

18. Top FH, Jr., Buescher EL, Bancroft WH, Russell PK. Immunization with live types 7 and 4 adenovirus vaccines. II. Antibody response and protective effect against acute respiratory disease due to adenovirus type 7. J Infect Dis. 1971;124(2):155-60.

19. Dudareva M, Andrews L, Gilbert SC, Bejon P, Marsh K, Mwacharo J, et al. Prevalence of serum neutralizing antibodies against chimpanzee adenovirus 63 and human adenovirus 5 in Kenyan children, in the context of vaccine vector efficacy. Vaccine. 2009;27(27):3501-4.

©Oxford University

20. Nwanegbo E, Vardas E, Gao W, Whittle H, Sun H, Rowe D, et al. Prevalence of neutralizing antibodies to adenoviral serotypes 5 and 35 in the adult populations of The Gambia, South Africa, and the United States. Clinical and diagnostic laboratory immunology. 2004;11(2):351-7.

21. Casimiro DR, Chen L, Fu TM, Evans RK, Caulfield MJ, Davies ME, et al. Comparative immunogenicity in rhesus monkeys of DNA plasmid, recombinant vaccinia virus, and replication-defective adenovirus vectors expressing a human immunodeficiency virus type 1 gag gene. J Virol. 2003;77(11):6305-13.

22. Kobinger GP, Feldmann H, Zhi Y, Schumer G, Gao G, Feldmann F, et al. Chimpanzee adenovirus vaccine protects against Zaire Ebola virus. Virology. 2006;346(2):394-401.

23. Xiang Z, Gao G, Reyes-Sandoval A, Cohen CJ, Li Y, Bergelson JM, et al. Novel, chimpanzee serotype 68-based adenoviral vaccine carrier for induction of antibodies to a transgene product. J Virol. 2002;76(6):2667-75.

24. Morris SJ, Sebastian S, Spencer AJ, Gilbert SC. Simian adenoviruses as vaccine vectors. Future virology. 2016;11(9):649-59.

25. Xiang Z, Li Y, Cun A, Yang W, Ellenberg S, Switzer WM, et al. Chimpanzee adenovirus antibodies in humans, sub-Saharan Africa. Emerging infectious diseases. 2006;12(10):1596-9.

26. Colloca S, Barnes E, Folgori A, Ammendola V, Capone S, Cirillo A, et al. Vaccine vectors derived from a large collection of simian adenoviruses induce potent cellular immunity across multiple species. Sci Transl Med. 2012;4(115):115ra2.

27. Afolabi MO, Tiono AB, Adetifa UJ, Yaro JB, Drammeh A, Nebie I, et al. Safety and Immunogenicity of ChAd63 and MVA ME-TRAP in West African Children and Infants. Molecular therapy : the journal of the American Society of Gene Therapy. 2016;24(8):1470-7.

28. Kimani D, Jagne YJ, Cox M, Kimani E, Bliss CM, Gitau E, et al. Translating the immunogenicity of prime-boost immunization with ChAd63 and MVA ME-TRAP from malaria naive to malaria-endemic populations. Molecular therapy : the journal of the American Society of Gene Therapy. 2014;22(11):1992-2003.

29. Tapia MD, Sow SO, Lyke KE, Haidara FC, Diallo F, Doumbia M, et al. Use of ChAd3-EBO-Z Ebola virus vaccine in Malian and US adults, and boosting of Malian adults with MVA-BN-Filo: a phase 1, single-blind, randomised trial, a phase 1b, open-label and double-blind, dose-escalation trial, and a nested, randomised, double-blind, placebo-controlled trial. The Lancet Infectious diseases. 2016;16(1):31-42.

30. Vellinga J, Smith JP, Lipiec A, Majhen D, Lemckert A, van Ooij M, et al. Challenges in manufacturing adenoviral vectors for global vaccine product deployment. Human gene therapy. 2014;25(4):318-27.

31. Dicks MD, Spencer AJ, Edwards NJ, Wadell G, Bojang K, Gilbert SC, et al. A novel chimpanzee adenovirus vector with low human seroprevalence: improved systems for vector derivation and comparative immunogenicity. PLoS One. 2012;7(7):e40385.

32. Chartier C, Degryse E, Gantzer M, Dieterle A, Pavirani A, Mehtali M. Efficient generation of recombinant adenovirus vectors by homologous recombination in Escherichia coli. J Virol. 1996;70(7):4805-10.

33. Havenga M, Vogels R, Zuijdgeest D, Radosevic K, Mueller S, Sieuwerts M, et al. Novel replication-incompetent adenoviral B-group vectors: high vector stability and yield in PER.C6 cells. The Journal of general virology. 2006;87(Pt 8):2135-43.

34. Vitelli A, Folgori A, Scarselli E, Colloca S, Capone S, Nicosia A. Chimpanzee adenoviral vectors as vaccines – challenges to move the technology into the fast lane. Expert Review of Vaccines. 2017;16(12):1241-52.

35. Folegatti PM, Bellamy D, Roberts R, Powlson J, Edwards NJ, Mair CF, et al. Safety and Immunogenicity of a Novel Recombinant Simian Adenovirus ChAdOx2 as a Vectored Vaccine. Vaccines. 2019;7(2).

©Oxford University

36. Pembrey L, Raynor P, Griffiths P, Chaytor S, Wright J, Hall AJ. Seroprevalence of cytomegalovirus, Epstein Barr virus and varicella zoster virus among pregnant women in Bradford: a cohort study. PLoS One. 2013;8(11):e81881.

37. Louie RE, Dobkin MB, Meyer P, Chin B, Roby RE, Hammar AH, et al. Measurement of rabies antibody: comparison of the mouse neutralization test (MNT) with the rapid fluorescent focus inhibition test (RFFIT). Journal of biological standardization. 1975;3(4):365-73.

©Oxford University

Appendix 3: Laboratory Adverse Event Severity Grading

THE JENNER INSTITUTE
DEVELOPING INNOVATIVE VACCINES

UNIVERSITY OF OXFORD

Appendix A: Oxford Laboratory Adverse Event Severity Grading

(Ox) Routine Haematology			Lab Range	Grade 0	Grade 1	Grade 2	Grade 3
Haemoglobin	**Male**	g/l	130 - 170	126 - 170	115 - 125	100 - 114	<100
	**Female**		120 - 150	114 - 150	105 - 113	90 - 104	<90
White Blood Cells	**Elevated**	x109/l	4.00 - 11.00	3.51 - 11.49	11.50 - 15.00	15.01 - 20.00	>20
	**Low**				2.50 - 3.50	1.50 - 2.49	<1.50
Platelets		x109/l	150 - 400	136 - 400	125 - 135	100 - 124	<100
Neutrophils		x109/l	2.0 - 7.0	1.5 - 7.0	1.00 - 1.49	0.50 - 0.99	<0.50
Lymphocytes		x109/l	1.0 - 4.0	1.0 - 4.0	0.75 - 0.99	0.50 - 0.74	<0.50
Eosinophils		x109/l	0.0 - 0.5	0.0 - 0.64	0.65 - 1.50	1.51 - 5.00	>5.00

Routine Biochemistry			Lab Range	Grade 0	Grade 1	Grade 2	Grade 3
Sodium	**Elevated**	mmol/l	135 - 145	135 - 146	147 - 148	149 - 150	>150
	**Low**				132 - 134	130 - 131	<130
Potassium	**Elevated**	mmol/l	3.5 - 5.0	3.4 - 5.0	5.1 - 5.2	5.3 - 5.4	>5.4
	**Low**				3.2 - 3.3	3.0 - 3.1	<3.0
Urea		mmol/l	2.5 - 7.4	2.5 - 8.1	8.2 - 8.9	9.0 - 11.0	>11.0
					1.1-1.5*ULN	>1.5-3.0*ULN	>3.0*ULN
Creatinine		µmol/l	49 - 104	49 - 113	114 -156	157 - 312	>312
					1.3-1.5*ULN	>1.5-2.0*ULN	>2.0*ULN
Bilirubin	**Normal LFT**	µmol/l	0 - 21	0 - 26	27 - 31	32-42	>42
					1.1-1.25*ULN	>1.25-1.5*ULN	>1.5-1.75*ULN
Bilirubin	**Abormal LFT**	µmol/l	0 - 21	0 - 22	23 - 26	27 - 31	>31
When accompanied by any increase in LFT							
					1.25 - 2.5*ULN	>2.5 - 5.0*ULN	>5.0*ULN
ALT		IU/l	10 - 45	10 - 55	*56 - 112*	*113 - 225*	*>225*
					1.1 - 2.0*ULN	>2.0 - 3.0*ULN	>3.0*ULN
Alk Phosphatase		IU/l	30 - 130	30 - 142	*143 - 260*	*261 - 390*	*>390*
Albumin		g/l	32 - 50	32 - 50	28 - 31	25 - 27	<25

Coagulation Studies		Lab Range	Grade 0	Grade 1	Grade 2	Grade 3
				1.10*ULN	>1.10 - 1.20*ULN	>1.2*ULN
Prothrombin time	s	9.0 - 12.0	9.0 - 13.1	13.2	13.3 - 14.4	>14.4
(APTT)				1.10 - 1.20*ULN	>1.20 - 1.40*ULN	>1.4*ULN
Activated Partial Thromboblasin Time	s	20.0 - 30.0	20.0 - 32.9	33.0 - 36.0	36.1 - 42.0	>42.0
Fibrinogen	g/l	1.5 - 4.0	1.5 - 4.0	1.25 - 1.49	1.0 - 1.24	<1.0

Other		Lab Range	Grade 0	Grade 1	Grade 2	Grade 3
AST				1.25 - 2.5*ULN	>2.5 - 5.0*ULN	>5.0*ULN
Aspartate transferase	IU/l	15 - 42	15 - 51	*52 - 105*	*106 -210*	*>210*
GGT				1.25 - 2.5*ULN	>2.5 - 5.0*ULN	>5.0*ULN
Gamma-glytamyl transpeptidase	IU/l	15 - 40	15 - 50	*51 - 100*	*101 - 200*	*>200*

VC027 Appendix (OUH - v3.0 27 Jul 2016)

Severity grading criteria for clinically significant laboratory abnormalities; adapted from FDA guidelines (1) using Oxford University Hospitals NHS Foundation Trust laboratory reference ranges.
